# Mechanochemistry of Metal Hydrides: Recent Advances †

**DOI:** 10.3390/ma12172778

**Published:** 2019-08-29

**Authors:** Jacques Huot, Fermín Cuevas, Stefano Deledda, Kaveh Edalati, Yaroslav Filinchuk, Thierry Grosdidier, Bjørn C. Hauback, Michael Heere, Torben R. Jensen, Michel Latroche, Sabrina Sartori

**Affiliations:** 1 Hydrogen Research Institute, Département de Chimie, Biochimie et Physique, Université du Québec à Trois-Rivières, Trois-Rivières, QC G9A 5H7, Canada; 2 Institut de Chimie et des Matériaux Paris Est (UMR7182), CNRS, UPEC, F-94320 Thiais, France; 3 Department for Neutron Materials Characterization, Institute for Energy Technology, P.O. Box 40, NO-2027 Kjeller, Norway; 4 WPI, International Institute for Carbon-Neutral Energy Research (WPI-I2CNER), Kyushu University, Fukuoka 819-0395, Japan; 5 Institute of Condensed Matter and Nanosciences, Université catholique de Louvain, Place L. Pasteur 1, 1348 Louvain-la-Neuve, Belgium; 6 Laboratoire d’Étude des Microstructures et de Mécanique des Matériaux (LEM3), Université de Lorraine, CNRS UMR 7239, 7 rue Félix Savart, BP 15082, CEDEX 03, 57073 Metz, France; 7 Institute for Applied Materials—Energy Storage Systems (IAM-ESS), Karlsruhe Institute of Technology Hermann-von-Helmholtz-Platz 1, 76344 Eggenstein-Leopoldshafen, Germany; 8 Interdisciplinary Nanoscience Center (iNANO) and Department of Chemistry, University of Århus, Langelandsgade 140, DK-8000 Århus C, Denmark; 9 Department of Technology Systems, University of Oslo, NO-2027 Kjeller, Norway

**Keywords:** Mechano-synthesis, cryomilling, high-pressure torsion, cold rolling, metal hydrides, reactive ball milling, surface mechanical attrition, in-situ characterization.

## Abstract

This paper is a collection of selected contributions of the *1st International Workshop on Mechanochemistry of Metal Hydrides* that was held in Oslo in May 2018. In this paper, the recent developments in the use of mechanochemistry to synthesize and modify metal hydrides are reviewed. A special emphasis is made on new techniques beside the traditional way of ball milling. High energy milling, ball milling under hydrogen reactive gas, cryomilling and severe plastic deformation techniques such as High-Pressure Torsion (HPT), Surface Mechanical Attrition Treatment (SMAT) and cold rolling are discussed. The new characterization method of in-situ X-ray diffraction during milling is described.

## 1. Introduction

Since the pioneering work of Benjamin, mechanochemical treatment has been extensively used to produce new materials or to enhance properties of existing alloys [1,2]. In the field of metal hydrides, the most extensively used method has been ball milling. Very interesting and important results were achieved by using ball milling to synthesize and modify metal hydrides. However, other techniques should be considered as they could bring other types of effect on the metal hydrides and also may be more appropriate for industrial applications.

From an historical point of view, metal or complex hydrides for energy storage applications were synthesized by solvent-based or by direct solid–gas hydrogenation reactions. Although these techniques are important, during the past two decades, advanced mechanochemistry has become increasingly dominating. Traditionally, “ball milling” was used as a grinding technique, e.g., to provide homogenous well mixed powder samples with reduced particle size for solid-state reactions as well as for mechanical alloying of metals [3]. Mechanochemistry was considered limited to alloying reactions, by “random mixing of atoms”.

Today, more advanced techniques are developed, which combine preparation of reactive precursors for solvent-based synthesis and mechanochemistry [4]. Nevertheless, the technique has advanced significantly since the usage in “grinding mode”. Today, the ball milling mode is associated with mechanochemistry. It is one of the most widely accepted and easily employable techniques for materials scientists, as no Schlenk line manipulations are required [5].

Solid-state chemists widely use “ball milling” for the synthesis and preparation of novel metal hydrides as new materials for energy storage and battery technologies and, with that, for new solutions within “sustainable green chemistry”, i.e., solvent free synthesis methods [3,6,7,8].

Mechanochemical synthesis can be conducted under a range of different conditions, such as cryogenic temperatures, under inert or reactive atmosphere, or at elevated gas pressures or in a solvent, and/or with high-impact energy between the reacting compounds [9,10,11]. Hence, mechanochemistry is a very versatile technique, which can be employed for preparation of a wide range of novel materials [5].

Hydrogen has an extremely diverse chemistry and forms compounds with most elements in the periodic table. Mechanochemistry has been widely used to prepare new types of hydrides and has significantly expanded the variety of compositions and structures of these materials. Initially, the interest was focused on solid state hydrogen storage [12,13,14,15], mainly based on magnesium hydride [16,17,18]. Mechanochemistry has provided a variety of new composite samples and knowledge of additives effect and alloying reactions. In the past two decades, a wide range of complex metal hydrides have been synthesized using mechanochemistry [19]. These compounds are very interesting new classes of energy materials for batteries and hydrogen storage applications [20,21,22].

This review presents the latest achievements in the field using high-energy ball milling, cryomilling, milling under hydrogen pressure, and milling while investigation via in situ diffraction. In addition, other mechanochemical methods are discussed. This is justified from a fundamental as well as a practical point of view. Fundamentally, other techniques could bring different characteristics to the processed alloys, for example texture, low or high angle grain boundaries, different types of defects, etc. From a practical point of view, other techniques beside ball milling should be investigated for large scale applications. Recently, Severe Plastic Deformation (SPD) techniques such as High-Pressure Torsion (HPT) and Cold Rolling (CR) have been investigated for processing metal hydrides. In the following, the effect on metal hydrides synthesis and performances of various mechanochemical techniques are presented and discussed.

## 2. High-Energy Mechanochemical Synthesis

Deeper knowledge to the chemistry in the “black box”, i.e., the closed ball milling vial, has facilitated advanced utilization of high-energy mechanochemistry as an adequate tool for material synthesis. The products obtained with this approach may often be impossible to prepare by standard solvent-based methods. Moreover, “over-milling” of less stable materials often leads to “destructive chemical reactions” and to the synthesis of decomposition products. This section introduces the experimental approach of high-energy ball milling along with selected examples, challenges and pitfalls, which need to be considered. We add a detailed discussion of which reactions may take place in the vial during mechanochemical treatment.

### 2.1. The Planetary Ball Mill

The planetary ball mill is the focus in this section due to its versatility described below. Nevertheless, there are types of ball mills with even higher impact energies such as the SPEX shaker mills. Advanced planetary ball mills, such as the Fritsch Pulverisette (FRITSCH GmbH) No. 4, 5 or 6 are widely used. And the Pulverisette No. 4 consists of a main disk and two secondary disks, called planets, which have named the apparatus. Vials containing the reaction mixture and a defined number of balls are mounted on the top of the planets. The balls and vial are ideally made or coated with the same material, e.g., steel, tungsten carbide (WC), aluminum oxide (Al_2_O_3_) or zirconium oxide (ZrO_2_). These materials have different density, hardness and other mechanical properties, which explains why it is best to use the same material during milling to avoid gradients in hardness and, therefore, avoid destructive forces on one of the materials.

The vials can be loaded in an inert atmosphere inside a glove box. Typical ball-to-powder mass ratio is between ~10:1 and ~35:1. The lower is the ratio, the lower is the ball-to-ball and ball-to-vial friction. Thus, a higher ratio favors a higher contamination of the sample with impurities from balls and vial material, combined with the hardness and mechanical properties of balls and vial. Modifications in experimental parameters may compromise the purity of the obtained products.

The main disk and the planets are rotating in opposite directions, and the planets rotate reversely usually at twice the speed of the main disk. Typical speeds are between 200 and 400 rpm, causing the balls to move and fly inside the vials. The Fritsch Pulverisette No. 4 allows the speed of the planets, ω, to be varied independently of the main disk speed, Ω [23]. In fact, the relative rotation speed, ω/Ω, during mechanochemical treatment determines the trajectory of the balls inside the vial [24]. The ball trajectories also depend on the mill geometry, i.e., the ratio between the main disk radius, *R*, and the vial radius, *r*. The ball trajectories can be used to subdivide the approach in two regimes: (a) the traditional grinding mode where the balls follow the circumference of the vial; and (b) the high-energy mode with ball trajectories approaching the center of the vial. The main focus here is on the latter high-energy regime.

The ball trajectories through the center of the vial produce large impact energies mainly between balls and vial wall. This energy can be further optimized using a vial with limited height and larger diameter. However, ball material density, ball diameter (i.e., mass of balls), ball-to-powder ratio also determine the impact energy. The impact energy can be calculated according to Refs. [25,26] and are further discussed in Ref. [3]. The high energies produced by collisions of the balls and vial walls indicates that few larger balls facilitate high-impact energies rather than many smaller balls.

The variation of the main disk speed, Ω, is necessary to obtain different milling energies, i.e., higher rotational speeds provide larger impact energies of the balls on the powder material in the vial. Furthermore, the larger main disk speed provides a larger number of impacts per unit time and, thereby, increases the total amount of energy transferred to the powder [3]. However, the higher energy transferred to the material along with the unavoidable frictional energy will heat up the material and temperatures higher than 100 °C may readily be achieved. This, often unwanted, heating effect may be limited by intervening the mechanochemical treatment by pauses, e.g., 2–5 min milling followed by 2–5 min pause, etc.

### 2.2. Solvent Free Synthesis of Metal Borohydrides

Metal borohydrides are well known as a reducing agent in organic chemistry [27]. The first mechanochemical synthesis of metal borohydride was shown in 1953 for the synthesis of sodium borohydride from boric oxide and sodium hydride (see Reaction (1)) [28,29].

4NaH + 2B_2_O_3_ → NaBH_4_ + 3NaBO_2_ (1)

A comprehensive overview of the borohydrides and their derivatives can be found in Refs. [30,31].

Initially, the preferred procedure for synthesizing *RE* borohydrides was the “solvent-free” approach, where a *RE*-chloride and an alkali metal borohydride (Li, Na, and K) are reacted in a one-step mechanochemical synthesis [31,32]. Thus far, LiBH_4_ has proven to be the most efficient precursor during these reactions, possibly due to formation of the stable reaction product LiCl [31,32,33,34,35,36,37,38,39,40,41,42,43,44,45,46,47,48]. However, other alkali metal borohydrides, i.e., NaBH_4_ and KBH_4_, have been employed as well as rare earth metal hydrides [49].

*Metathesis reaction.* The above-mentioned examples mainly utilize the metathesis, or double substitution reaction. A classic example is the synthesis of Y(BH_4_)_3_ from the reactants YCl_3_−LiBH_4_ (1:3), which also results in the formation of the unwanted product LiCl, according to Reaction (2) [36,37].

YCl_3_ + 3LiBH_4_ → Y(BH_4_)_3_ + 3LiCl (2)

The polymorph α−Y(BH_4_)_3_ was previously obtained by a solvent-based method in diethyl ether using solutions of LiBH_4_ and YCl_3_ at room temperature (RT), but this polymorph was also obtained by ball milling with varying amounts of another new polymorph, β−Y(BH_4_)_3_ [36,37]. α−Y(BH_4_)_3_ is considered the stable polymorph at ambient conditions, whereas β−Y(BH_4_)_3_, is assumed to be a high-pressure polymorph, but the β-polymorph is also stable at RT [35,36,37,50]. The β-polymorph is suggested to form by a α- to β-Y(BH_4_)_3_ polymorphic transition due to the high pressure during mechanochemical treatment.

*Addition reaction.* Another established but less often observed reaction is the addition. This is illustrated by the synthesis of KZn(BH_4_)Cl_2_ obtained by mechanochemical treatment of a mixture of ZnCl_2_ and KBH_4_ (1:1) [5,51], as shown in Reaction (3).

ZnCl_2_ + KBH_4_ → KZn(BH_4_)Cl_2_ (3)

It is highly interesting to note that the new compound KZn(BH_4_)Cl_2_ contains the heteroleptic complex ion [Zn(BH_4_)Cl_2_]^−^ where Zn coordinates to two chloride ions and two hydrogen atoms in η^2^−BH_4_, CN(Zn) = 4 [51].

The addition reaction, Reaction (3), clearly create a material with a fully ordered crystal structure, hence, contrasts the synthesis of the solid solution shown in Reaction (4).

(1−x)LiBH_4_ + xLiCl → Li(BH_4_)_1_−_x_Cl_x_ (4)

*Complex mechanochemical reactions*. Mechanochemistry has provided a series of new lithium ion conductors, Li*RE*(BH_4_)_3_Cl, *RE* = Ce, La, Gd, which likely cannot be prepared by any other method [52]. Initially, a new compound, LiCe(BH_4_)_3_Cl, was discovered in a mechanochemically treated sample of CeCl_3_–LiBH_4_ (1:3). The complex reaction is believed to be a coupled metathesis and addition reaction, described by Reactions (5) and (6), below.

CeCl_3_ + 3LiBH_4_ → Ce(BH_4_)_3_ + 3LiCl (5)

Ce(BH_4_)_3_ + LiCl → LiCe(BH_4_)_3_Cl (6)

The system LaCl_3_–LiBH_4_ can be used to illustrate the complexity of the mechanochemical synthesis as ball milling of this mixture (1:6) results in an *addition reaction* and a product of LiLa(BH_4_)_3_Cl [44,53].

Mechanochemical synthesis experiments in the ZnCl_2_−MBH_4_ system clearly proceeds via more complex chemical reactions [54].

2ZnCl_2_ + 5LiBH_4_ → LiZn_2_(BH_4_)_5_ + 4LiCl (7)

2ZnCl_2_ + 5NaBH_4_ → NaZn_2_(BH_4_)_5_ + 4NaCl (8)

ZnCl_2_ + 3NaBH_4_ → NaZn(BH_4_)_3_ + 2NaCl (9)

Reactions (7)–(9) are assumed to be complex combinations of metathesis and addition reactions. Surprisingly, Reactions (8) and (9) provide different products despite being conducted at the same mechanochemical conditions, but only with small differences in composition of reactants. These observations tend to suggest that a state of chemical equilibrium occurs during mechanochemical synthesis. Mechanochemistry of reactants with dominantly ionic and covalent bonding clearly can lead to distinct chemical reactions and are not a statistical distribution of reactants as expected, e.g., for mechanical alloying of metals.

**Figure 1:**
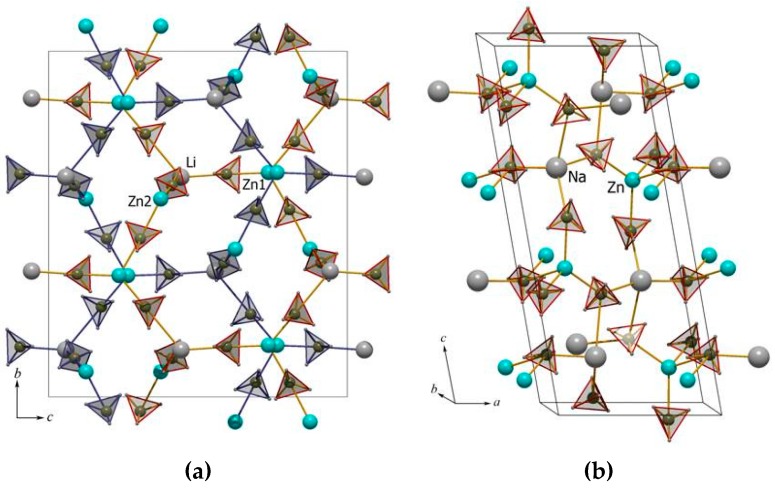
Crystal structure of MZn_2_(BH_4_)_5_, M = Li or Na: (**a**) built from isolated complex anions, [Zn_2_(BH_4_)_5_] and NaZn(BH_4_)_3_; and (**b**) consisting of a single three-dimensional network, containing [Zn(BH_4_)_3_]^−^ anions [54,55].

The structural topology of NaZn(BH_4_)_3_ and MZn_2_(BH_4_)_5_, M = Li or Na, are significantly different, possibly owing to differences in the mechanisms in Reactions (7)–(9). Interestingly, the structures of MZn_2_(BH_4_)_5_, M = Li or Na, are isostructural and built from two identical interpenetrated 3D frameworks consisting of isolated complex anions, [Zn_2_(BH_4_)_5_]^−^. This type of structural motif is known from open-structured molecular frameworks, so-called metallic organic frameworks (MOF). The compound NaZn(BH_4_)_3_ consists of a single three-dimensional network, containing [Zn(BH_4_)_3_]^−^ (see Figure 1) [54,55] and, thus, reveals the complexity of mechanochemistry.

*Coupled chemical reactions*. Reactions (7)–(9) illustrate the general drawback of metathesis related reactions. It shows that ionic compounds may contaminate the products—most often binary alkali metal halides but in some cases also ternary halides. Formation of LiZn_2_(BH_4_)_5_ in Reaction (7) proceeds completely, i.e., with formation of LiCl as the only side product, in contrast to NaZn_2_(BH_4_)_5_ and NaZn(BH_4_)_3_, Reactions (8) and (9), which only proceeds partly due to presence of a simultaneous and competing reaction forming a ternary metal chloride (see Reaction (10)).

ZnX_2_ + 2MX → M_2_ZnX_4_ (10)

This type of side reactions seems to be more pronounced the heavier the halide and alkali metal elements are, e.g., the ease of formation order of: Na_2_ZnBr_4_ > Na_2_ZnCl_4_ > Li_2_ZnCl_4_ [56]. These three compounds are prepared mechanochemically using stoichiometric mixtures of MX and ZnX_2_. However, formation of minor amounts of M_2_ZnX_4_ suggests that Reaction (10) is only weakly coupled with the formation of the metal borohydrides, i.e., Reactions (8) and (9) are faster than Reaction (10).

Side reaction may also be strongly coupled to the reaction forming the product, which has strong consequences for composition of the product and the optimal reactant composition. An illustrative example is the synthesis of alkali metal scandium borohydrides, NaSc(BH_4_)_4_ and KSc(BH_4_)_4_, by mechanochemistry from the reactants ScCl_3_ and NaBH_4_ or KBH_4_ (Reactions (11) and (12)) [57,58].

ScCl_3_ + 4NaBH_4_ → NaSc(BH_4_)_4_ + 3NaCl (11)

ScCl_3_ + 4KBH_4_ → KSc(BH_4_)_4_ + 3KCl (12)

However, alkali metal halides were not detected by powder X-ray diffraction in the product of any of the ball-milled samples of ScCl_3_−MBH_4_ (M = Na or K) in molar ratios 1:2, 1:3 or 1:4. This suggests that other, faster, reactions take place simultaneously, which consume the formed alkali metal halide, and indeed a new ternary alkali scandium chloride M_3_ScCl_6_ was identified. Surprisingly, the ScCl_3_−MBH_4_ (1:2) samples show diffraction from neither ScCl_3_ nor MBH_4_ and appear to contain the largest fraction of products NaSc(BH_4_)_4_ and KSc(BH_4_)_4_ [57,58]. Furthermore, the sample ratios ScCl_3_−MBH_4_ (1:3) and (1:4) contain different amounts of MBH_4_ but no diffraction from ScCl_3_. These observations are assigned to an addition reaction, which is responsible for the formation of Na_3_ScCl_6_ and K_3_ScCl_6_ (see Reactions (13) and (14)).

ScCl_3_ + 3NaCl → Na_3_ScCl_6_ (13)

ScCl_3_ + 3KCl → K_3_ScCl_6_ (14)

Assuming that the reactions for the formation of the ternary salts, M_3_ScCl_6_, Reactions (13) and (14) are much faster as compared to the formation of the metal borohydrides, MSc(BH_4_)_4_ (see Reactions (11) and (12)). Therefore, the Reactions (13) and (14) are strongly coupled to Reactions (11) and (12), respectively. Such reactions can be added together to form a reaction scheme for the overall reactions (see Reactions (15) and (16)). These reactions reveal that the optimal ratio for formation of MSc(BH_4_)_4_ and M_3_ScCl_6_ is in fact ScCl_3_−MBH_4_ (1:2) and the maximum yields of NaSc(BH_4_)_4_ and KSc(BH_4_)_4_ were 22 and 18 wt%, respectively.

2ScCl_3_ + 4NaBH_4_ → NaSc(BH_4_)_4_ + Na_3_ScCl_6_ (15)

2ScCl_3_ + 4KBH_4_ → KSc(BH_4_)_4_ + K_3_ScCl_6_ (16)

The lithium system ScCl_3_−LiBH_4_ did not show any formation of ternary metal halides and one reaction scheme explains the formation of LiSc(BH_4_)_4_ and LiCl [57,58]. Table 1 summarizes selected mechanochemical reactions.

### 2.3. Solvent Free Synthesis of “Reactive Hydride Composites”

Reactive hydride composites (RHC) are a combination of materials following an approach to destabilize metal hydrides by the formation of multicomponent hydride mixtures and, thereby, lowering the stability or reaction enthalpy. This was first described by Reilly and Wiswall with the example of MgH_2_ + MgCu_2_ formation upon absorption, and Mg_2_Cu + H_2_ upon desorption [81]. The advantage of RHC is the possibility to conserve the high hydrogen content of the original materials while anion substitution may occur but decrease the hydrogen capacity only slightly.

In the early 2000s, Chen et al. described the RHC including LiNH_2_ and LiH (Reaction (17)).

LiNH_2_ + 2 LiH → Li_2_NH + LiH + H_2_ → Li_3_N + 2H_2_ (17)

In the following years, further RHC were investigated including MgH_2_ + NaBH_4_, MgH_2_ + LiBH_4_ and MgH_2_ + Ca(BH_4_)_2_ [82,83,84,85]. It was discovered that MgB_2_, as a thermal reaction product, is crucial for rehydrogenation and offers a pathway for the formation of new borohydrides [82,83,84,86,87]. Eventually, a possibility to hydrogenate 2LiH and MgB_2_ including a catalyst (2–3 mol% TiCl_3_) following Reaction (18) was presented [82].

2LiBH_4_ + MgH_2_ → 2LiH + MgB_2_ + 4H_2_ (18)

This RHC revealed a reduction in enthalpy from 67 kJ mol^−1^ of H_2_ for the LiBH_4_/LiH and B system to 42 kJ mol^−1^ of H_2_, therefore improving thermodynamic properties compared to pure LiBH_4_ immensely [82].

Further efforts have been made in investigating NaH + MgB_2_, with a theoretical hydrogen capacity of 7.8 wt% for a 2:1 molar equivalent. The suggested reaction products upon hydrogenation are 2NaBH_4_ and MgH_2_, but an intermediate reaction product, NaMgH_3_, is formed, which decreases the hydrogen capacity substantially. Furthermore, the reaction is plagued with slow hydrogen sorption kinetics, meaning that 1 h is required to absorb 3.8 wt% H at 50 bar hydrogen and 400 °C, and this is the maximum experimentally obtained hydrogen absorption capacity [88,89,90,91]. To improve kinetics and absorption capacity, the approach of anion substitution and the influence of long-time milling has been investigated [92]. By substituting 10 mol% of NaH by NaF and forming the RHC of NaF + 9NaH + MgB_2_, reaction pathway in Reaction (19) is expected upon hydrogenation.

NaF + 9NaH + 5MgB_2_ + 20H_2_ → 10NaBH_3.9_F_0.1_ + 5MgH_2_ (19)

With a nominal hydrogen capacity of 7.7 wt%, the hydrogen capacity is slightly decreased compared to the original RHC (7.8 wt%). Additionally, the effect of ball milling was investigated with milling times of 87 h (long milled) and 5 h (short milled). These two samples absorb 6.0 and 6.3 wt% H, respectively. Therefore, the uptake of hydrogen is almost doubled compared to the original investigation under the reported conditions. However, a different hydrogenation pathway, with the products being NaBH_4_, MgH_2_ and NaMgH_3−x_F_x_ (0 ≤ x ≤ 1), is observed. The thermodynamics are modified for the temperature of formation of NaBH_4_, which decreases from 380 °C [91] to 206 °C in the long milled RHC. In a previous investigation, NaMgH_3_ was observed before the formation of NaBH_4_, which is contrary to the reported findings with formation NaBH_4_ before NaMgH_3−x_F_x_. Curiously, in the long milled RHC, NaBH_4_ and NaMgH_3−x_F_x_ reaction products form in greater amounts (~40 wt%), while, in the short milled system, under the same conditions, those products only appeared in minor amounts (~10 wt%). For the latter, it is assumed that a hydrogen containing, but purely scattering, boron rich phase (“B_48_”) is responsible for the lack of NaBH_4_ and NaMgH_3−x_F_x_ [90].

### 2.4. Challenges, Pitfalls and Deliberate Destructive Mechanochemistry

Although mechanochemical synthesis is a widely used technique, there are challenges, pitfalls and certain aspects to consider when it comes to its destructive use. Traditional ball milling conducted in the “grinding mode” is often a continuous process lasting over many hours and may lead to significant heating of particles. With this high-energy method, it is possible to synthesize stable as well as metastable compounds [3]. To further emphasize the challenges of steel equipment and the continuous process of milling, it is worth noting that induced impurities of Fe can be as high as 4 wt% after extended milling intervals [92]. Materials such as WC coated vials and WC balls are used to avoid iron contamination. Nevertheless, as these are very brittle, contamination is again unavoidable.

The observations from “grinding mode” may lead to the misconception that mechanochemistry is “just” random mixing of elements, but in truth, it has become clear that complex reactions often take place. However, prolonged mechanochemical treatment in the “grinding mode” may lead to decomposition of the sample and has in fact been utilized to develop novel “deliberate destructive mechanochemistry” synthesis strategies. A concept being illustrated by synthesis of nanosized vanadium boride in a matrix of halides, which are readily dissolved and removed by water [93]. It also led to the development of a new approach to synthesize anion-substituted sodium chloride [93,94,95].

In several cases, a metal chloride and an alkali metal borohydride are mechanochemically treated. The reaction product reveals clear diffraction from the alkali metal chloride and some minor unidentified diffraction peaks, e.g., for the synthesis of Sc(BH_4_)_3_ [96], V(BH_4_)_2_ and Cr(BH_4_)_2_ [97,98,99,100]. This suggests that the sample was “over-milled” or that the synthesis product was amorphous. A mechanochemical synthesis of Zn(BH_4_)_2_ was also reported [96,101], but the diffraction data did not allow for determination of the structure. Nonetheless, the synthesis product appears to be a mixture of NaZn_2_(BH_4_)_5_ and NaZn(BH_4_)_3_ [54,55].

#### 2.4.1. Reaction Byproduct

Meanwhile, the solvent-based synthesis of metal borohydrides has been employed for over 50 years [102]. The advantage of this method is the ability to remove the byproducts (e.g., LiCl) to yield pure products, thus allowing for the accurate determination of their physical properties [32,44,103,104]. The detriment of the solvent-based synthesis is the possible decomposition of, for instance the *RE* borohydride upon removal of the solvent [105]. On the contrary, mechanochemical synthesis does allow for the facile synthesis of these materials without complicated in vacuo manipulations and possible decomposition of the desired product [102].

#### 2.4.2. LiBH_4_ and the Influence of its Deliberate Destructive Decomposition Product

Previous methods for the synthesis of trivalent rare earth (*RE*) metal borohydrides are based on the reaction of LiBH_4_ with *RE*Cl_3_ [106,107]. The products obtained are reported to be contaminated with amorphous LiBH_4_ as the metathesis reaction of LiBH_4_ and *RE*Cl_3_ does not go to completion. Instead of a complete conversion into *RE*(BH_4_)_3_ and LiCl, it is plausible that some LiBH_4_ is among the reaction products. LiBH_4_ in minor amounts can be hard to observe utilizing laboratory powder X-ray diffraction (PXD) due to its weak scattering or possibly even amorphous phases.

The presence of amorphous LiBH_4_ has consequences for the thermal properties and chemical reactivity of samples prepared. For example, La(BH_4_)_3_ synthesized according to the aforementioned method forms Li_3_K_3_La_2_(BH_4_)_12_ upon the reaction with KBH_4_ [108]. Therefore, the focus has been on new synthetic strategies and investigations of chemical, physical and structural properties of the pure compounds. A new method to obtain solvate complexes, *RE*(BH_4_)_3_S(CH_3_)_2_, *RE* = Pr, Nd, and the corresponding borohydrides, *RE*(BH_4_)_3_, *RE* = Pr, Nd, allows detailed investigation of the polymorphic transformations [109]. Although a solvate-based synthesis is reported, the activation step of the samples remains high-energy mechanochemical milling.

An intriguing detail for the understanding of the influence of amorphous LiBH_4_ has been reported and explains a rehydrogenation pathway of an erbium borohydride composite [47]. Recent reports have shown that in pure Er(BH_4_)_3_, synthesized by mechanochemical milling of ErCl_3_+ LiBH_4_, up to 20% of the initial released hydrogen could be rehydrogenated [110]. Although an explanation of reaction products was not given, it was shown later that pure Er(BH_4_)_3_, from a solvent-based synthesis, does not reabsorb hydrogen, e.g., at 400 °C and 100 bar H_2_. Nevertheless, by mixing 50 wt% LiH into a desorbed sample of Er(BH_4_)_3_ and applying similar rehydrogenation conditions as described above, ErH_3_ and LiBH_4_ were formed [47].

Summarizing, amorphous LiBH_4_ in minor amounts are impossible to observe in PXD but its decomposition product LiH, is most likely the starting point for the rehydrogenation pathway reported in Ref. [47]. A RHC using this knowledge, showed that Er(BH_4_)_3_ with additional LiH and LiBH_4_ can have very decent rehydrogenation behavior of 88% and 83% after the second and third desorption/absorption cycles, respectively [111].

#### 2.4.3. Conclusions

To finalize this section, all aforementioned investigations indicate that pressure induced transitions as well as composition are of major importance, in particular for high-energy ball milling. Ball milling can induce high pressure and mechanical stress between the reacting materials. Thus, facilitating chemical reactions, in contrast to traditional solid-state synthesis techniques, which are mainly temperature “driven”, i.e., reactions occur due to cation diffusion in the solid state [112]. Previously, ball milling was mainly devoted to the preparation of alloys and solid solutions of metals, but nowadays comprises ionic, ionic/covalent and organic molecules. In some cases, novel materials are only accessible through mechanochemistry and, apparently, cannot be obtained by other means.

## 3. Cryomilling—Mechanical Processing and Synthesis at Low Temperatures

Cryomilling is used for mechanochemical processing and synthesis of materials at low temperatures. In a SPEX Freezer/Mill, the vial with powder and a stainless-steel impactor is placed in liquid nitrogen (77 K). The impactor is the only moving part, and it moves back and forth by an oscillating electromagnetic field. The powder is trapped between the impactor and the ends of the vial each time a collision occurs. The impact frequency is up to 30 Hz.

When ductile metals/materials are cryomilled, the low temperature will promote embrittlement of the ductile phase, the cold welding to the milling media is limited and there will be a limited deviation from the nominal composition. The increased embrittlement at low temperature will also lead to reduced particle sizes and shortened diffusion paths. Cryomilling is for example used to process Mg-based alloys [113,114]. Furthermore, cryomilling is important for synthesis of metastable compounds/phases since the heat released in the wanted reaction may be absorbed by the vial before critical temperatures for thermal decomposition are reached. This implies that, compared to room temperature, the metastable compounds are less likely to decompose due to reduced mobility. One example is synthesis of the unstable borohydride LiZn_2_(BH_4_)_5_ [115]. Another important example is the synthesis of aluminum hydride AlH_3_ (alane), and here we review and present new results of properties of AlH_3_ synthesized by cryomilling.

AlH_3_ has a high gravimetric (10.1 wt%) and volumetric hydrogen density (148 g H_2_/L). Hydrogen can be released at moderate temperatures, which makes the compound interesting for hydrogen storage applications. AlH_3_ has been found to take at least six different crystal structures depending on the synthesis route: α, α’, β, γ, δ and ε [116,117,118,119,120,121,122]. AlH_3_ is metastable at ambient conditions, decomposing into aluminum metal and hydrogen gas at 60–80 °C [123,124,125]. This reaction is not reversible at moderate conditions; H_2_ pressure larger than 2.5 GPa is required to rehydride Al powder.

**Figure 2:**
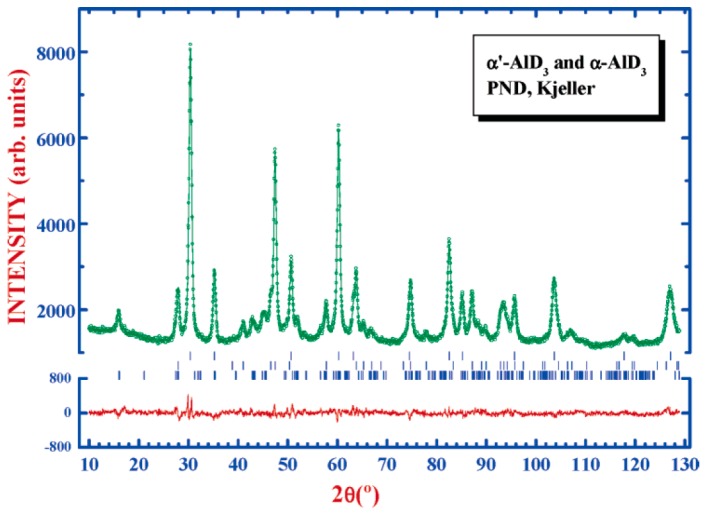
Observed intensities (circles) and calculated intensities from Rietveld refinements (upper line) of cryomilled 3LiAlD_4_ + AlCl_3_ at RT for powder neutron diffraction (PUS, JEEP II, and IFE) data. Positions of Bragg reflections are shown with bars for LiCl, α-AlD_3_, and α’-AlD_3_ (from top). The difference between observed and calculated intensities is shown with the bottom line. Taken from [117].

AlH_3_ polymorphs have traditionally been synthesized by organometallic methods. Since AlH_3_ is not thermally stable at room temperature (RT), dehydrogenation during ball milling at ambient conditions must be avoided. When 3LiAlD_4_ + AlCl_3_ are ball milled in a planetary ball mill at RT, small amounts of α- and α’-AlD_3_ were detected along with LiCl and Al [117]. By cryomilling 3LiAlD_3_ + AlCl_3_ in a SPEX Freezer/Mill, significant amounts of α- and α’-AlD_3_ together with LiCl were detected, and the quantitative phase analysis indicated a mixture of 34% α’-AlD_3_ and 66% α-AlD_3_ [117] (see Figure 2).

Cryomilling the mixture 3NaAlH_4_ + AlCl_3_ gave an increased total amount of alanes compared to the synthesis based on 3LiAlD_3_ + AlCl_3_: the ratio α’/α was increased to 1.04, but the synthesized alane has lower thermal stability [126]. The ratio of polymorphs can to some extent be controlled by adding additives and seed crystals. Small amounts of FeF_3_ (isostructural to β-AlH_3_ and α’ is similar to the β polymorph ) added to 3LiAlD_4_ + AlCl_3_ increased the α’/α ratio to 1.05 [126]. α-AlF_3_ is isostructural to α-AlH_3_, and cryomilling 3 mol% of AlF_3_ with β-AlD_3_ gave a significant decrease of the α’/α ratio, and thus highest amount of α-AlD_3_ [127]. Additional of TiF_3_ lead to full decomposition to Al.

Since α-AlH_3_ takes the same structure as α-AlF_3_ and α’-AlH_3_ is isostructural to β-AlF_3_, it has been investigated experimentally, by ab initio calculations and thermodynamic modeling, if fluorine anion substation can take place in AlH_3_ [128]. The samples were prepared by cryomilling. However, fluorine substitution does not seem feasible for the alane system, and thus not similar to F-substitution in Na_3_AlH_6_ [129].

To assess the potential of cryomilled AlH_3_ for hydrogen storage applications, tests were recently carried out with two aims: (i) gain information about reactivity and influence of passivation layers (mainly oxides) on the decomposition behavior of alane; and (ii) study the metastability of alane after partial decomposition.

Both issues are crucial for application purposes. On the one hand, it is important to know if impurities due to air exposure will cause a degradation of the material’s performance or change its characteristics. On the other hand, it is of interest to know if the decomposition process continues after the temperature of the system is lowered below the onset temperature for thermal desorption.

Reactivity and passivation tests were carried out on two different sets powders: (i) as-prepared (cryomilled) alane heated from RT to 400 °C; and (ii) cryomilled alane exposed to air for 24 h and heated from RT to 400 °C.

**Figure 3:**
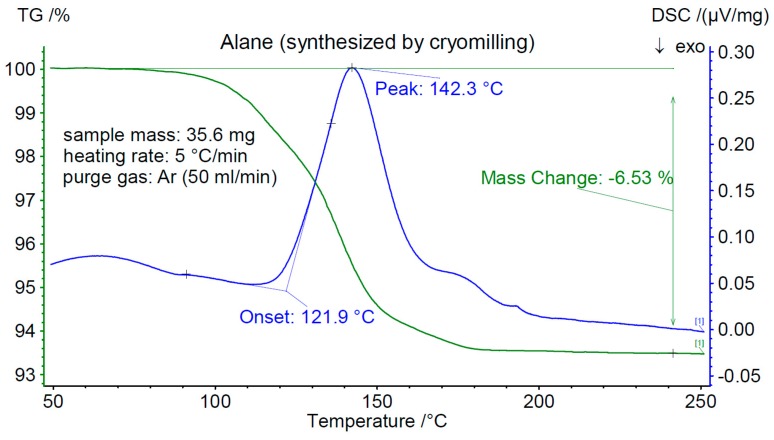
DSC-TGA data of a cryo-milled sample. Data are shown between RT and 250 °C.

**Figure 4:**
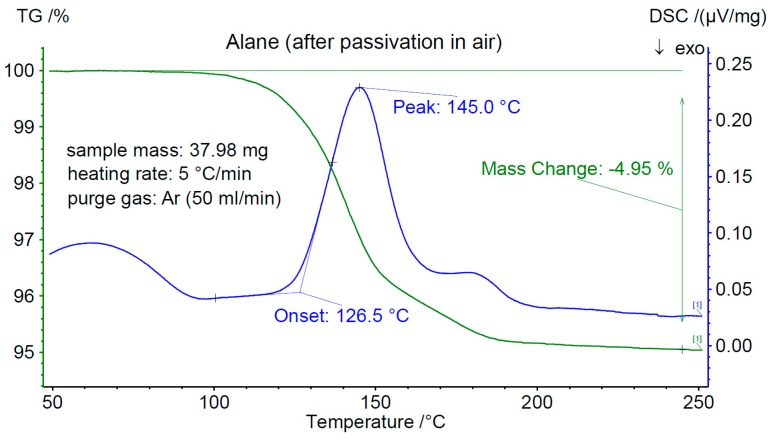
DSC-TGA data of a passivated sample (exposed to air for 24 h). Data are shown between RT and 250 °C.

The results of the passivation test are summarized in Figure 3 and Figure 4, which display the DSC and TGA traces for the two samples between 25 and 250 °C. The as-prepared cryomilled material (Figure 3) loses about 6.5 wt% of its storage capacity in the temperature region 25 < *T* < 250 °C. In this case, the onset temperature for desorption, *T_on_*, was determined to be 121(1) °C. A different cryo-milled sample passivated by air exposure for 24 h (Figure 4) shows a weight loss of about 5 wt% between RT and 250 °C and displays a *T_on_* of 126(1) °C. The shift and decrease in capacity could be explained by the formation of oxides (hydroxides) which create a surface barrier that needs to be overcome.

The reactivity tests were carried out in a fume hood by exposing as-milled and passivated powder to air. No combustion was observed after ca. 1 min of air exposure. Dripping water (H_2_O) on the air-exposed powder produced mild bubbling.

The metastability of cryomilled AlH_3_ was investigated by DSC-TG and by in situ synchrotron radiation powder X-ray diffraction (SR-PXD). The aim of these measurements was to test whether the desorption reaction can be controlled—i.e., stopped—if the temperature is decreased from the onset temperature for desorption down to RT.

The as-synthesized cryomilled alane powder was heated with a constant heating rate (5 K/min) at three different temperatures: at the onset temperature for desorption *T_on_*; 10 K above the onset temperature *T_on+10K_*; and 10 K or 15 K below the onset temperature *T_on-10K_* or *T_on-15K_*, respectively.

DSC-TG isotherms carried out on the three samples at RT right after heating (not shown) show that the signal of the thermobalance stays constant over the duration of the isothermal measurement. This clearly indicates that there is no sign for a continuation of the desorption process after the temperature has been lowered to RT. This suggests that alane could be safely stored and that the decomposition can be fully controlled by lowering the temperature below the onset temperature, e.g., when the system is in standby mode.

**Figure 5:**
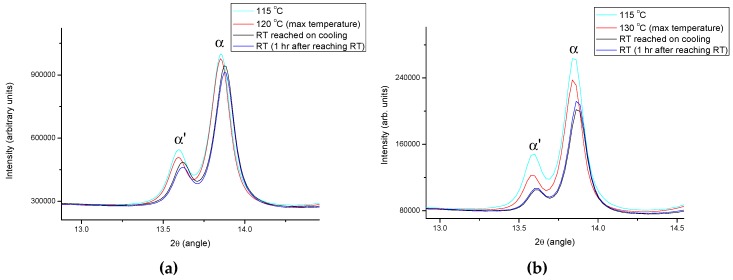

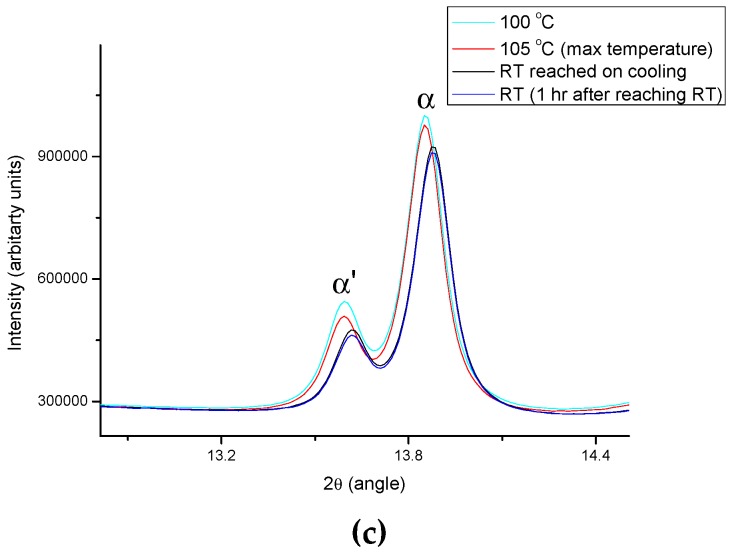
Bragg peaks from α- and α’-AlH3 during in-situ SR-PXD decomposition after heating at: (**a**) T_on_; (**b**) T_on+10K_; and (**c**) T_on-15K_ and cooling to RT.

Synchrotron radiation power X-ray diffraction (SR-PXD) measurements were performed at the Swiss–Norwegian Beamlines (SNBL) at the European Synchrotron Radiation Facility (ESRF) in Grenoble, France. Decomposition reactions of alane in closed boroglass capillaries, heated with a hot air blower, were followed in situ. A diffraction pattern was collected every 30 s. As expected, the RT SR-PXD data (not shown) revealed a mixture of the phases α- and α’-AlH_3_, LiCl and Al. Heating the material from ambient temperature with a rate of 5 K/min, the onset of decomposition was found to be between 117 and 120 °C, from where the alane peaks diminish and the Al peaks increase in intensity. Figure 5a–c shows the reduction of the Bragg peak intensities of α- and α’-AlH_3_ after heating at *T_on_*, *T_on+10K_*, and *T_on-15K_*.

As expected, the reduction is larger for the materials after heating 10 K above the *T_on_* (Figure 5a). There is no significant further decrease in the AlH_3_ peaks after dwelling 1 h at RT. It is worth pointing out that a reduction of the AlH_3_ peaks is also observed for the sample heated to 15 K below *T_on_* (Figure 5c), thus indicating that some desorption takes place also below the onset temperature determined by heating with 5 °C/min. Furthermore, there was no appreciable change during the 1-h isotherm at RT.

In summary, the tests described above clarified that the reactivity of the powder when exposed to air is limited and passivation in air does not change significantly the reactivity, while it lowers the amount of hydrogen released during desorption. Furthermore, the decomposition process of AlH_3_ stops when the temperature of the system is lowered below the onset temperature for thermal desorption. The latter finding is technologically relevant for potential applications in solid-state hydrogen storage.

## 4. Formation and Defect Generation of Lightweight Hydrides by Mechanochemistry under Hydrogen Gas

Mechanochemistry under hydrogen gas is a versatile synthesis route for the formation of hydride compounds. Indeed, many families of compounds, such as binary and ternary metal hydrides as well as Mg- and Al-complex hydrides, can be directly synthesized by mechanochemistry of their constituting metals under hydrogen gas [5]. Hydride formation is achieved for compounds that are thermodynamically stable at the pressure (*P*) and temperature (*T*) conditions at which the solid gas reactions occur inside the milling jar. As these experiments are performed at high pressure and temperature and with vials under mechanical stress, it is important to have certified equipment that meets all safety regulations.

The concept of mechanochemistry under pressure is supported by the work of Zhang et al. [130] showing the relevance of (*P*, *T*) local conditions in mechanochemistry. They studied the Mg-Si-H system by reactive ball milling starting from 2Mg, and Si powders as reactants under a hydrogen pressure of 9 MPa. Depending on the milling energy (tuned by changing the rotation speed of the vials), they obtained two different final equilibrium states, either Mg_2_Si or 2MgH_2_ + Si at high and low rotation speeds, respectively. Such comportment was interpreted by the change of the local temperature which increases with milling speed at the milling impact.

Beside the synthesis of stable hydride phases, whose formation can also be attained by the classical route of thermally driven solid–gas reaction, mechanochemistry under hydrogen gas opens the way to the formation of metastable phases thanks to severe plastic deformation effects induced by mechanical energy. Recent studies on the formation of lightweight imide and amide Li-(Mg)-N-H starting from Li_3_N (and Mg) as initial solid reactants are reviewed here [131,132]. These compounds deserve high interest as hydrogen storage materials [133,134] as well as Li-ion conductors [135,136,137].

Mechanochemistry of commercial Li_3_N under hydrogen gas (*P_H2_* = 9 MPa) entails the consecutive formation of lithium imide (Li_2_NH) and lithium amide (LiNH_2_) according to the reaction scheme given by Reactions (20) and (21) [132]:

Li_3_N + H_2_ → Li_2_NH + LiH (20)

Li_2_NH + LiH + H_2_ → LiNH_2_ + 2LiH (21)

**Figure 6:**
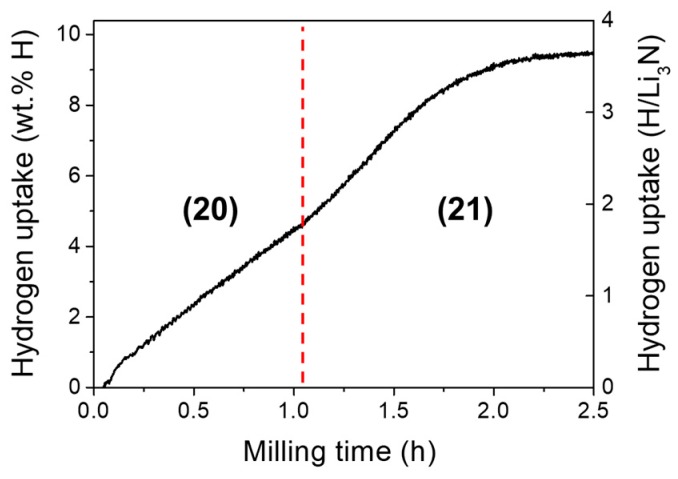
In situ hydrogen absorption during the mechanochemistry of Li_3_N powder under hydrogen gas (*P_H2_* = 9 MPa). The vertical dashed line shows the discontinuity in the uptake curve that reflects the two reaction Reactions (20) and (21) associated with the formation of lithium imide (left) and amide (right) compounds. Reproduced from Ref. [137] with permission from the PCCP Owner Societies.

As shown in Figure 6, the hydrogen uptake curve monitored during mechanochemistry reveals that the full hydrogenation is accomplished in less than 2.5 h of milling time *t_m_* and consists in two steps. Hydrogen uptake is linear during the first step. This has been attributed to the constant rate of mechanical collisions. X-ray dffraction analysis showed that the first step comprises the transformation of the polymorph α-Li_3_N (S.G. *P*6/*mmm*) into the β-Li_3_N (S.G. *P*6_3_/*mmc*) metastable phase and the reaction of the latter with hydrogen to form lithium imide (Reaction 20). High local mechanical pressure at impact between α-Li_3_N powder and milling tools lead to the formation of β-Li_3_N, which is the stable polymorph of Li_3_N at *P* > 0.6 GPa [138]. The metastable conditions of β-Li_3_N are close to those of the high-pressure orthorhombic γ-MgH_2_ polymorph (stable phase at *P* > 2 GPa [139]), which has been widely observed during mechanical milling of magnesium hydride [140,141]. As for the second reaction step (Reaction 21), leading to lithium amide formation, the hydrogen uptake curve has a sigmoidal shape. This is characteristic of Avrami‘s nucleation and growth processes [142]. Moreover, a detailed kinetic analysis based on solid-state models indicates that amide formation is controlled by a one-dimensional Li-vacancy mechanism [132]. Structural data support this analysis and evidence formation of mixed imide/amide Li_2−*x*_NH_1+*x*_ compounds as intermediates. Lithium imide and amide phases formed by mechanochemistry under hydrogen gas crystallize in the low nanocrystalline range with crystal size of 10 ± 2 nm.

Phase and structural evolution during mechanochemistry of Li_3_N under hydrogen gas shows clear differences as compared to the thermally driven solid–gas reaction. First, in the mechanochemical route, formation of intermediate phase Li_4_NH at low hydrogen content is not observed. This “quasi-imide” Li_4_NH phase has however been detected by in-situ neutron diffraction at the early stage of hydrogenation during thermally driven reaction at 250 °C [143] Second, the hydrogenation path by mechanochemistry at *P_H2_* = 9 MPa follows a two-step reaction (Reactions (20) and (21)), whereas, for the thermally-driven reaction (~200–250 °C), a single step is observed also using elevated pressures (*P_H2_* ≈ 1 MPa). The latter result has been evidenced in several in-situ neutron diffraction studies [144,145].

**Figure 7:**
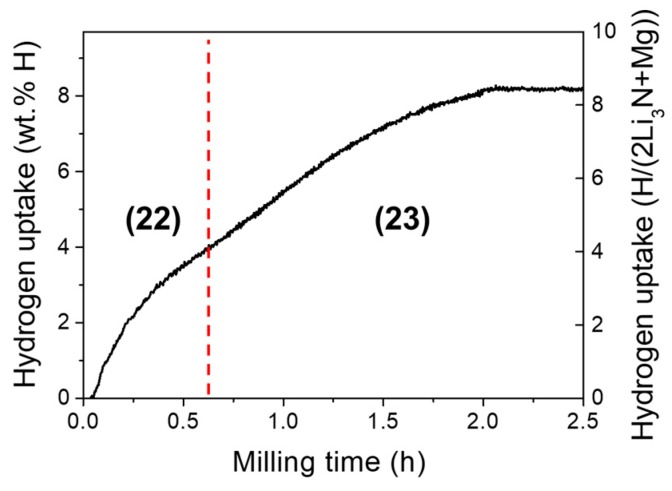
In situ hydrogen absorption during the mechanochemistry of 2Li_3_N+Mg mixed powder under hydrogen gas (*P_H2_* = 9 MPa). The vertical dashed line shows the discontinuity in the uptake curve that reflects the two reaction Reactions (22) and (23) associated with the formation of cation-mixed nitride/imide Li_3_MgN_2_H (left) and magnesium amide Mg(NH_2_)_2_ (right) compounds. Reprinted by permission from Springer, Ref. [146].

The addition of Mg to the Li-N-H system deserves particular interest since a high hydrogen amount (5.6 wt%) can potentially be reversibly stored at normal conditions of pressure and temperature in the Li-Mg-N-H system [134]. To gain access to this system by mechanochemistry, lithium nitride and magnesium in molar ratio 2:1 were milled under hydrogen gas. The in situ hydrogen uptake curve displayed in Figure 7 reflects, as in the previous case, two consecutive reactions leading to full absorption in *t_m_* ≈ 2 h [146]. By structural analysis of deuterated compounds [131], they were assigned to the reaction scheme:

2Li_3_N + Mg+ 2H_2_ → Li_3_MgN_2_H + 3LiH (22)

Li_3_MgN_2_H + 3LiH + 3H_2_ → Mg(NH_2_)_2_ + 6LiH (23)

**Figure 8:**
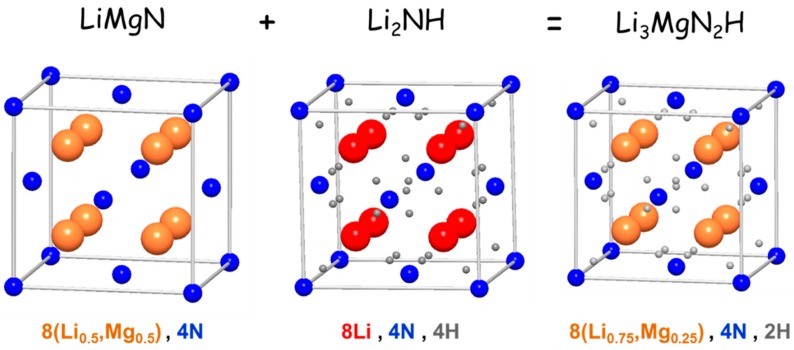
Crystal structure of cation-mixed nitride/imide Li_3_MgN_2_H as an intermixing of high temperature anti-fluorite-type polymorphs LiMgN and Li_2_NH.

The Reaction (22) leads to the formation of a cation-mixed nitride/imide Li_3_MgN_2_H compound. It crystallizes in anti-fluorite type structure (S.G. *Fm*-3*m*) with local coexistence of N^3−^ and [NH]^2−^ ions on a face-centered cubic lattice as well as random occupancy of Li and Mg cations at tetrahedral sites. In fact, as depicted in Figure 8, the structure of Li_3_MgN_2_H can be regarded as an intermixing of the crystal structures of high-temperature disordered polymorphs of anti-fluorite type LiMgN nitride and Li_2_NH imide. Such a high level of structural disorder suggests that Li_3_MgN_2_H is a metastable compound whose existence is driven by mechanical deformations induced by milling. The Reaction (23) leads to the formation of long-range disordered (i.e., amorphous form) magnesium amide Mg(NH_2_)_2_ compound. The metastable amorphous phase Mg(NH_2_)_2_ crystallizes by thermal annealing on heating above 425 K (tetragonal S.G. *I*4_1_/*acd*).

To summarize, mechanochemical synthesis of lightweight imide/amide Li-(Mg)-N-H compounds using Li_3_N (and Mg) and hydrogen as initial reactants is accompanied by severe defect generation: creation of vacancies, short-range and long-range atomic disorder and metastability of high-temperature or high -pressure phases. This leads to the formation of novel phases such as high-pressure polymorphs (β-Li_3_N), locally disordered compounds (Li_3_MgN_2_H) and long-range disorder compounds (amorphous Mg(NH_2_)_2_).

## 5. High-Pressure Torsion

**Figure 9:**
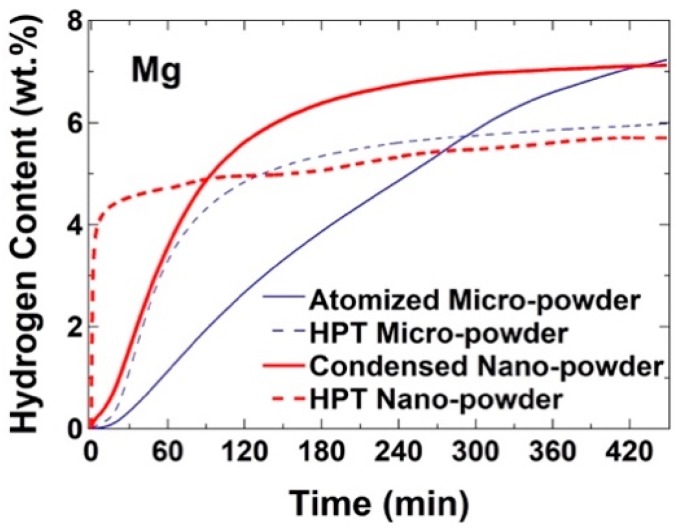
First hydrogen absorption kinetics measured at 673 K under a hydrogen pressure of 3.5 MPa for Mg micro-sized atomized powder and nano-sized condensed powder before and after HPT processing [160] (reproduced with permission).

High-pressure torsion (HPT) is currently one of the most popular severe plastic deformation (SPD) techniques to produce bulk nanostructured materials with a large fraction of lattice defects and enhanced functional properties [147,148]. In the HPT method, intense shear strain is induced in a bulk disc or powder sample by compressing the material between two anvils under high pressure and concurrent rotating the anvils with respect to each other [147,148]. Kosadume et al. in 2007 [149] conducted the first experimental study on the effect of HPT processing on the hydrogen storage properties. This study together with several other reports [150,151,152,153,154,155,156,157,158,159,160,161] confirmed the significance of HPT processing on improvement of hydrogen storage kinetics. As shown in Figure 9, this improvement in kinetics is sometimes accompanied by a reduction in hydrogen storage capacity in the first hydrogenation cycle [160]. The impact of HPT processing on hydrogen storage properties is not limited to fast kinetics, and the method can significantly enhance the activation and air resistivity, especially in difficult-to-activate Ti-based materials [159,162,163,164,165,166,167,168,169,170,171]. Moreover, the shearing nature of the deformation in HPT, together with enhanced defect-induced atomic diffusion, provide a unique opportunity for making composites [156,158] and for intermixing of elements to synthesize new phases even in the immiscible systems [166,172,173,174,175]. One advantage of the HPT processing, when applied on powder samples, is that the method is conducted in a very clean way compared to high-energy ball milling that usually involves some contamination from the atmosphere or tooling. Here, the significance of microstructural and structural features induced by HPT processing is discussed on hydrogen storage properties.

### 5.1. Significance of Grain Boundaries

**Figure 10:**
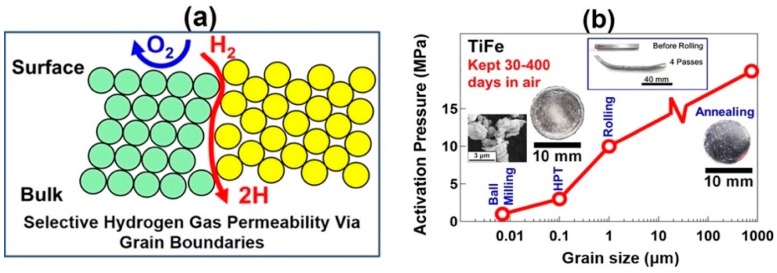
(**a**) Schematic illustration of activation mechanism by grain boundaries; and (**b**) variations of hydrogen activation pressure at room temperature against grain size for TiFe processed by annealing, groove rolling, HPT and ball milling [171] (reproduced with permission).

The best example of the effect of HPT on activation and air resistivity was reported for TiFe intermetallic and for Ti-based hydrogen storage materials [159,162,163,164,165,166,167]. TiFe was first introduced as a hydrogen storage material by Reilly and Wiswall in 1974 [176]. Although TiFe can thermodynamically store hydrogen at room temperature, its hydrogenation occurs only after an activation process at high temperature and/or high pressure [176]. Moreover, the thermally activated material is deactivated quickly once it is exposed to air because of intense surface oxidation. However, the HPT-processed TiFe can reversibly absorb and desorb hydrogen at room temperature without any activation treatment even after 400-day storage in air [159]. As shown in Figure 10a, it was suggested that the main mechanism for the HPT-induced activation is due to the formation of a large fraction of grain boundaries as pathways for hydrogen [162]. This mechanism was confirmed by further experiments showing that the activation pressure is reduced by the introduction of grain boundaries or by decreasing the grain size in samples processed by thermal annealing [159], grove rolling [163], HPT [159] and ball milling [177], as summarized in Figure 10b.

### 5.2. Significance of Dislocations

**Figure 11:**
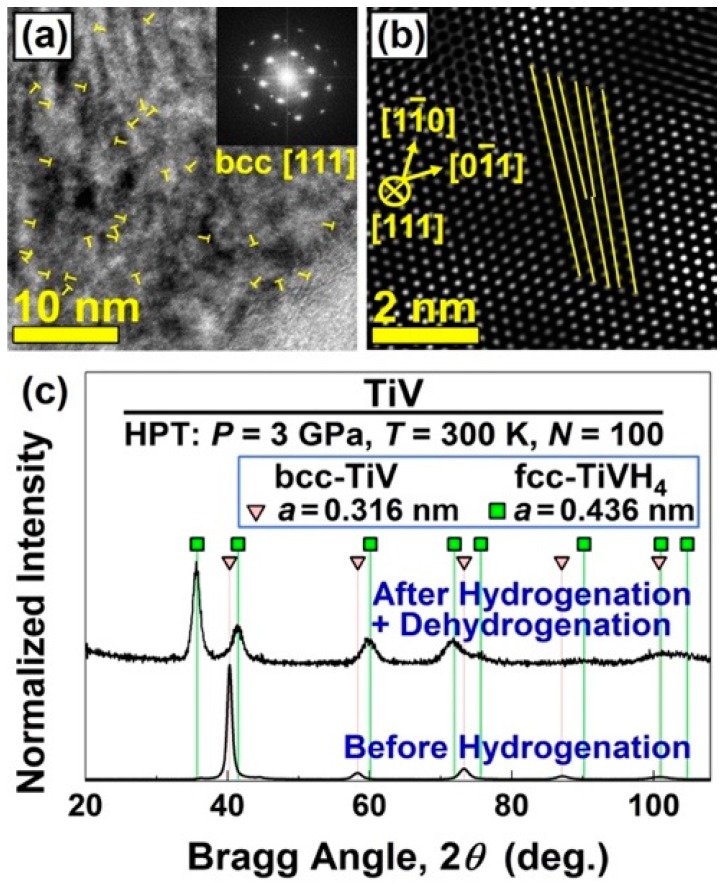
(**a**) TEM image of edge dislocations marked with T; (**b**) lattice image of a dislocation; and (**c**) XRD profile before and after hydrogenation and dehydrogenation at 303 K under 0.001–10 MPa hydrogen pressure for Ti-V alloy synthesized by HPT [166] (reproduced with permission).

When a conventionally fabricated as-cast sample is exposed to hydrogen, large fractions of dislocations are usually formed during the first hydrogenation cycles [178,179]. It was suggested that, in addition to the formation of powder particles (during pulverization), the formation of dislocations in the first cycles is one of the reasons that the material can uptake hydrogen more easily in the following cycles due to dislocation-assisted hydrogen transport and metal hydride phase transformations [178,179]. It is then expected that the introduction of dislocations in the initial materials can facilitate the hydrogen uptake. Images taken by transmission electron microscopy (TEM) presented in Figure 11 show the formation of a large fraction of dislocations in a HPT-synthesized Ti-V alloy with the bcc (body-centered cubic) structure [166].

Although the Ti-V alloys cannot usually absorb hydrogen without a sophisticated activation treatment [180,181], the HPT-processed Ti-V alloy with a large fraction of dislocation fully transforms to a TiVH_4_ hydride with the fcc (face-centered cubic) structure after two hydrogenation/dehydrogenation cycles at room temperature, as shown in the X-ray diffraction (XRD) pattern of Figure 11c. Here, it should be noted that the activation of Ti-V-based alloys by introduction of dislocations and grain boundaries can undesirably deteriorate their hydrogen storage reversibility [167], as discussed below.

### 5.3. Significance of Stacking Faults

**Figure 12:**
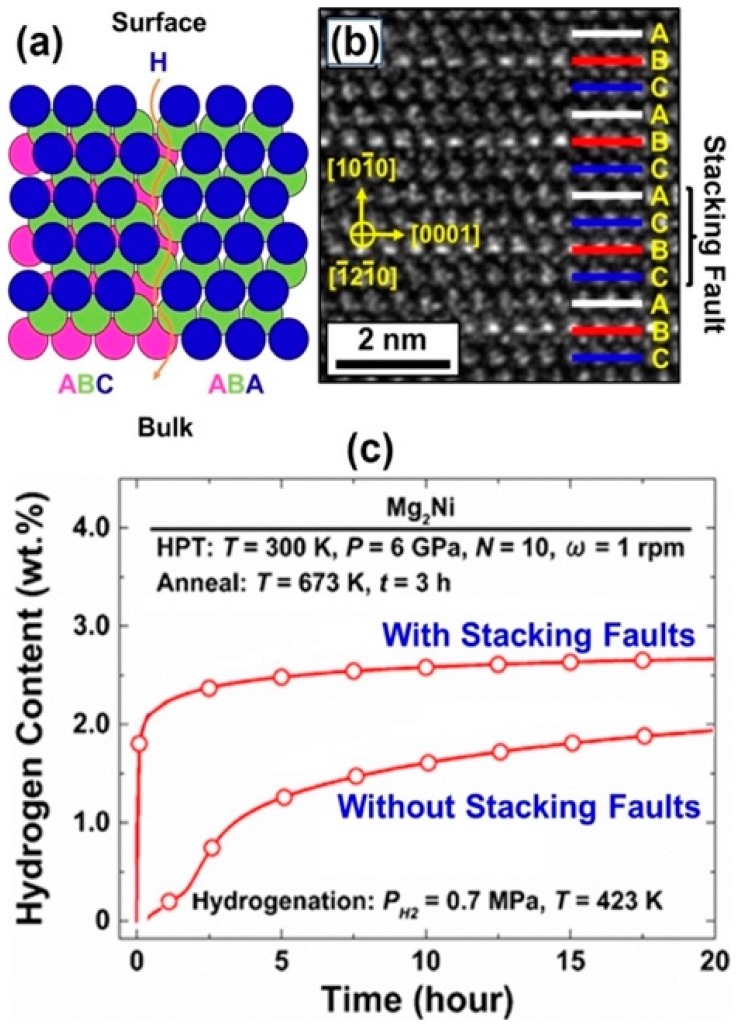
(**a**) Illustration of stacking fault effects as pathways for hydrogen; (**b**) TEM lattice image of a stacking fault in Mg_2_Ni after HPT processing followed by annealing; and (**c**) hydrogenation kinetic curves at 423 K under initial hydrogen pressure of 0.7 MPa for coarse-grained Mg_2_Ni with and without stacking faults [157] (reproduced with permission).

As shown in Figure 12a, stacking faults coupled with partial dislocations produce atomic pathways, which may be effective to transport hydrogen [157]. To investigate the effect of stacking faults on hydrogen storage performance, stacking faults were introduced in an Mg_2_Ni intermetallic by HPT processing followed by thermal annealing at 673 K [157]. Figure 12b shows a representative stacking fault compared with the ideal ABCABC stacking. As shown in Figure 12c while the coarse-grained sample with large fractions of stacking faults absorb 2 wt% of hydrogen within a few minutes, the sample without stacking faults exhibits very slow hydrogenation kinetics [157].

### 5.4. Significance of Localized Amorphization

**Figure 13:**
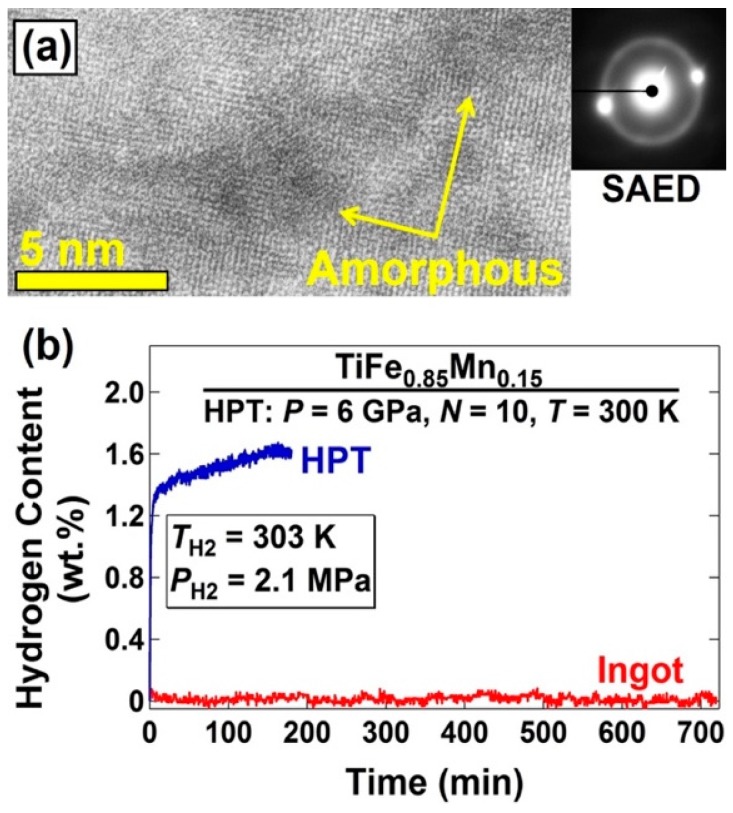
(**a**) TEM image and corresponding SEAD pattern with nano-sized amorphous regions indicated by arrows; and (**b**) hydrogenation kinetic curves for TiFe_0.85_Mn_0.15_ after ingot casting and HPT processing [165] (reproduced with permission).

Amorphous regions are considered as fully disordered regions and can act as pathways for hydrogen permeation [182]. In an attempt to activate the TiFe_0.85_Mn_0.15_ intermetallics [165], nano-sized amorphous regions were introduced by HPT processing in the materials, as shown in TEM lattice image and corresponding selected-area electron diffraction (SAED) pattern with a hallo ring in Figure 13a. As shown in Figure 13b, while the as-cast material does not absorb hydrogen at room temperature in agreement with earlier publications [183,184], partial amorphization by HPT leads to activation of material with fast hydrogenation kinetics. It should be noted that other studies showed that even amorphous alloys become more active with application of HPT processing because of the formation of glass/glass or glass/crystal boundaries [154,159]. For example, while the Mg_65_Ce_10_Ni_20_Cu_5_ amorphous alloy hardly absorb hydrogen after melt spinning, it becomes active after HPT processing and absorbs hydrogen at 393 K [159].

### 5.5. Significance of Phase Transformation

Severe shear straining, high pressure, supersaturated fraction of lattice defects and defect-induced atomic diffusion during HPT processing, make the method an effective tool to control phase transformations [147,148]. Emami et al. [174] showed that the HPT process combined with a subsequent thermal treatment is quite effective to synthesize a large variety of stable Mg-based intermetallics from elemental powder mixtures [174]. It was shown that the method is also effective to synthesize new metastable phases even from immiscible systems [170]. Table 2 summarizes formation of metastable phases from elemental powders in several systems by HPT processing. Among these metastable phases, a Mg_4_NiPd alloy with the CsCl-type crystal structure can reversibly absorb and desorb hydrogen at room temperature in good agreement with the predictions of first-principle calculations [168].

## 6. Surface Modification by Mechanical Attrition Treatment

Compared to an appreciable number of publications on the effect of SPD processing on hydrogen storage properties, there have been quite limited attempts to modify the hydrogen storage properties by surface treatment methods [167]. In most of the SPD methods, nanostructures with large fractions of lattice defects are generated all through the bulk [147]. However, in surface treatment methods, lattice defects and nanostructure are generated only on the surface either mechanically [185] or by beam-assisted processing [186]. Among various surface treatment techniques, the surface mechanical attrition treatment (SMAT) [187], which is also known as ultrasonic shot peening [188] or severe shot peening [189], has received significant attention to produce gradient structures (nanostructures on the surface and coarse grains in bulk) [187,190]. In this method, the surface is treated by metallic shots impacting the surface at velocities that can reach about 10 m/s [191,192]. It therefore bears some similarities with the high energy ball milling technique. However, the sample in SMAT is in the form of bulk plates which can be processed continuously without any limitation in the sample length. This makes the method appropriate for potential commercial applications. Moreover, as discussed below, the SMAT methods can be used as a scientific tool to have more insights into the role of surface defects and gradient structure on hydrogen storage properties.

### Significance of Surface Defects and Gradient-Structure

**Figure 14:**
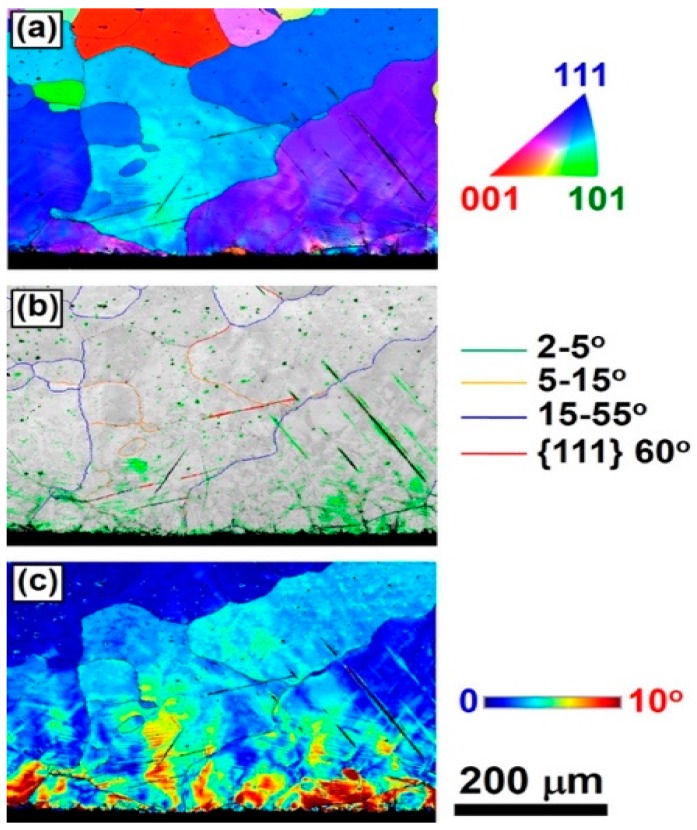
EBSD analysis of the gradient microstructure seeing on the cross section of a Ti_10_V_75_Cr_15_ after SMAT processing: (**a**) orientation map; (**b**) grain boundary distribution; and (**c**) internal misorientation. Treated surface is at bottom [167] (reproduced with permission).

In an attempt to understand the significance of gradient structures on hydrogen storage, two types of microstructure were produced in a Ti_10_V_75_Cr_15_ alloy with the bcc structure [167]. First, as illustrated by the electron back scattering diffraction (EBSD) analysis given in Figure 14, a gradient structure was produced by SMAT having a higher density of structural defects towards the surface (see large fraction of low angle grain boundaries and high internal misorientation on the treated surface in Figure 14b,c, respectively). Second, a uniform nanostructure all through the bulk was produced by HPT. Here, it should be noted that the Ti-V-Cr alloy was intentionally selected for this study because it can thermodynamically absorb and desorb ~2 wt% of hydrogen at room temperature [193], but it suffers from two drawbacks: (i) it needs a thermal treatment at high temperatures for initial activation [194,195]; and (ii) its reversibility is degraded with the introduction of lattice defects [196,197,198].

**Figure 15:**
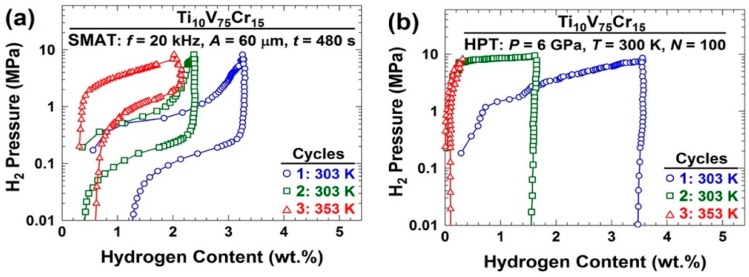
PCT isotherms at 303-353 K for Ti_10_V_75_Cr_15_ after: (**a**) SMAT; and (**b**) HPT [167] (reproduced with permission).

Figure 15 shows the pressure-composition-temperature (PCT) isotherms for samples processed by: (a) SMAT; and (b) HPT (PCT was conducted at 303 K for the first two cycles and at 353 K for the third cycle). Although Ti-V-Cr alloy after both SMAT and HPT processing interestingly absorbs hydrogen without any activation treatment, only the SMAT-processed alloy exhibits ~2 wt% hydrogen storage reversibility. The poor reversibility after HPT processing confirms that the presence of lattice defects such as dislocations and grain boundaries in the bulk can significantly degrade the reversibility of this specific alloy, in good agreement with some earlier reports [196,197,198].

**Figure 16:**
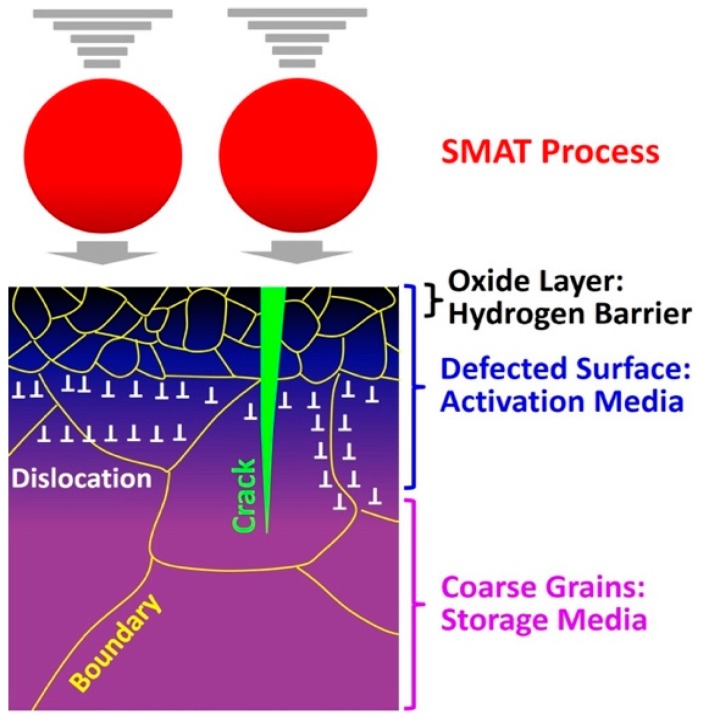
Mechanism of activation of Ti-V-Cr alloy with gradient structure after processing by SMAT [167] (reproduced with permission).

However, as schematically shown in Figure 16 for the gradient structure produced by SMAT, the lattice defects and cracks formed on the surface activate the materials by acting as pathways for hydrogen transport while the bulk of material—which still has coarse grains with lower density of lattice defects—can store hydrogen reversibly. The current results provide new insight into the importance of surface defects on activation.

## 7. Effect of Cold Rolling on Metal Hydrides

**Figure 17:**
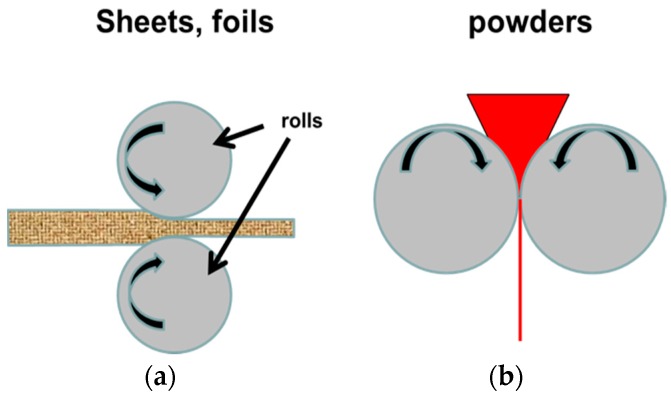
Schematic illustration of Cold Rolling (CR) configurations: (**a**) conventional; and (**b**) powder processing. Adapted from Ref. [199].

In the usual configuration, cold rolling is performed by introducing a metal plate between rollers where it is compressed and squeezed, as shown in Figure 17. The term cold rolling (CR) is used when the temperature of the metal is below its recrystallization temperature. For temperature higher than the recrystallization temperature, the term Hot Rolling should be used. As the vast majority of the investigations involving metal hydrides have been using CR, we restrict our discussion to this method. Cold rolling could be performed on the metal or on its hydride form. The problem with cold rolling a hydride is the brittle nature of most hydrides. Processing a powder in a horizontal milling machine is not convenient. A solution of this problem is to perform the milling vertically, as shown in Figure 17.

### 7.1. Cold rolling on Mg-Pd system

The effects of CR on magnesium and its alloys have been extensively studied [157,200,201,202,203,204,205,206,207,208,209,210,211,212,213,214,215,216,217,218,219,220,221,222,223,224,225,226,227]. One reason is that magnesium has a high hydrogen storage capacity (7.6 wt%). The other reason is that magnesium is ductile and could be processed easily by CR. However, as magnesium has a limited number of slip planes in its hexagonal crystal structure, it suffers an important work hardening upon repetitive CR [228,229]. Fortunately, for metal hydrides, this is usually not a problem because when hydrogenated the hydride will naturally turn into powder due to the large lattice expansion of the hydride phase.

The system Mg-Pd was the subject of some of the early studies on the effect of CR on magnesium alloys [201,202,206,207]. Because of the high price of palladium, this is not attractive for practical applications but, because palladium is a good catalyst and there are few intermetallics in the Mg-Pd phase diagrams, this system is a good candidate for fundamental investigation.

**Figure 18:**
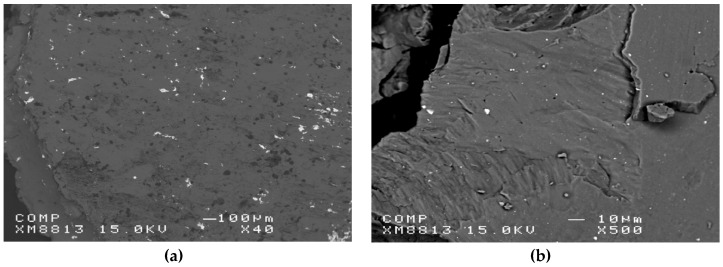
Backscattered electron micrographs of Mg–Pd 2.5 at%: (**a**) after 20 rolling passes; and (**b**) after 2 h of ball milling. The white marks are palladium particles. Adapted from Ref. [201].

Dufour and Huot have investigated the addition of a small amount of Pd by cold rolling. They used Mg plates of 1 mm thickness that were stacked with Pd foil of thickness 0.025 mm to get a proportion of 2.5 at% Pd [201]. Cold rolling was performed in air, at room temperature on a laboratory-scale rolling apparatus. After each roll, the sample was folded in two and rolled again thus having a 50% reduction of thickness at each rolling pass. Figure 18 shows the distribution of palladium in magnesium for samples rolled 20 times compared to the same composition milled 2 h on a SPEX8000 milling machine. The distribution of palladium is much coarser in the cold rolled sample compared to the ball milled one considering that the micrograph of the cold rolled sample is magnified 40 times while the magnification of the ball milled sample is 500×.

**Figure 19:**
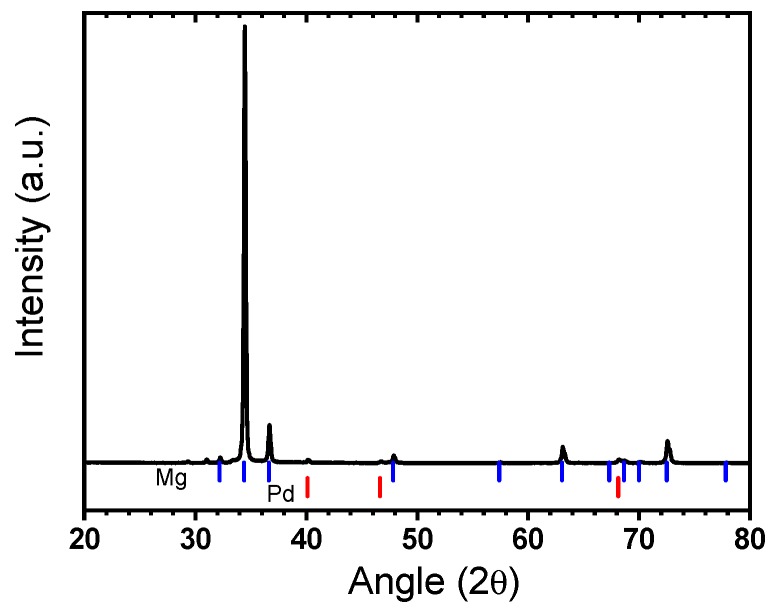
X-ray diffraction pattern of Mg–Pd 2.5 at% after 20 rolling passes. Cu kα radiation.

The X-ray diffraction pattern of the sample after cold rolling (20 passes) is shown in Figure 19. The sample is highly textured along (002) direction, which is expected for a hexagonal structure. The peaks are still relatively sharp, which indicates that the sample is not nanocrystalline. The palladium peaks are barely visible due to the low concentration of this element.

**Figure 20:**
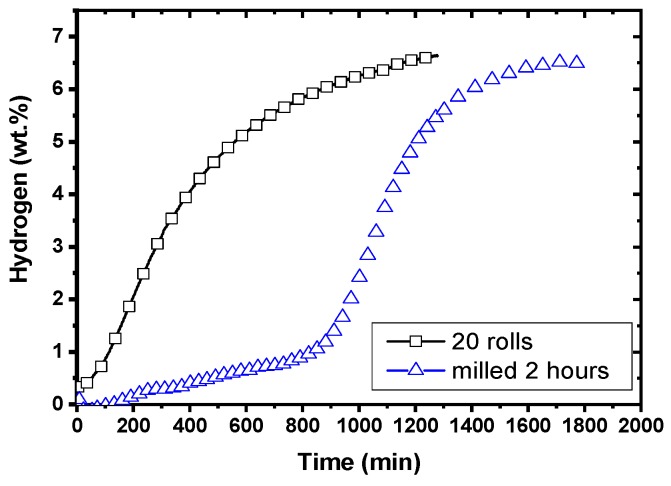
First hydrogenation at 623 K under 1.3 MPa of hydrogen of Mg–Pd 2.5 at% after 20 rolling passes and after 2 h of milling.

The first hydrogenation (activation) at 623 K and under a hydrogen pressure of 1.3 MPa is presented in Figure 20.

**Figure 21:**
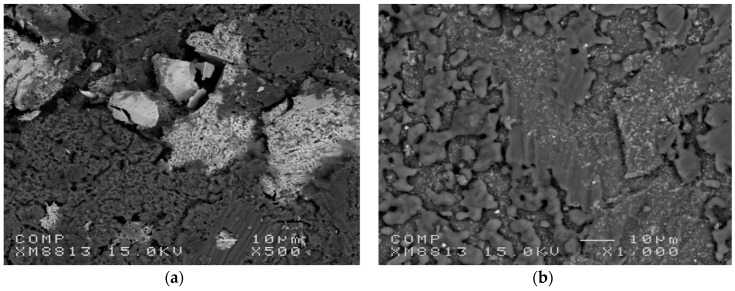
Backscattered electron micrographs of Mg–Pd 2.5 at% after first hydrogenation: (**a**) after 20 rolling passes; and (**b**) after 2 h of ball milling. The white areas are palladium-rich regions.

We see that the cold rolled sample has a much faster first hydrogenation compared to the ball milled one. The hydrogenation starts readily, without any incubation time as in the case of the ball milled sample. Figure 21 shows the backscatter micrographs of the cold rolled and ball milled samples after hydrogenation. In the case of cold rolled sample, the interdiffusion of magnesium and palladium results more important than for the ball milled sample. The cold rolled sample also seems to me more porous.

From these results, we conclude that even if the distribution of palladium is much finer in the ball-milled sample, the kinetic is not better than for the much coarser distribution of palladium. Therefore, particle size is not the only factor influencing the action of a catalyst. Cold rolling probably produces a much stronger connection between the magnesium matrix and the palladium particle. This facilitates the inter-diffusion of magnesium and palladium and produces a much faster kinetic.

### 7.2. Cold rolling on AB_5_ system

Many metal hydrides are ordered stoichiometric compounds formed by a hydride forming element (A) and a non-hydride forming element (B). The resulting ternary hydride could be written as A_m_B_n_H_x_ where m and n are integers and x is a real number [230]. Amongst the many different m and n combinations, the system AB_5_ is one of the most studied. The classical example is LaNi_5_, which was reported in 1970 by van Vucht et al. [231]. The AB_5_ type metal hydrides are mainly used as hydride electrodes in Ni-MH batteries but they are also considered for hydrogen storage and purification [232,233,234,235]. One of the problems facing the AB_5_ type metal hydrides is the slow first hydrogenation. This is usually explained by a thin surface oxide, which prevents hydrogen diffusion to the bare metal. Recently, it has been shown that cold rolling could greatly enhance the first hydrogenation of LaNi_5_ [236] and CaNi_5_ [237]. From the literature, the maximum capacity of CaNi_5_ and LaNi_5_ are, respectively, 1.8 wt% [238] and 1.5 wt% [231].

The LaNi_5_ powder and the raw CaNi_5_ alloy (99.5% pure) were both used in their as-received state. A Durston DRM 130 cold rolling apparatus was used but it was modified in order to be able to perform cold rolling vertically instead of horizontally. Details of the cold rolling and ball milling are given in Ref. [236].

**Figure 22:**
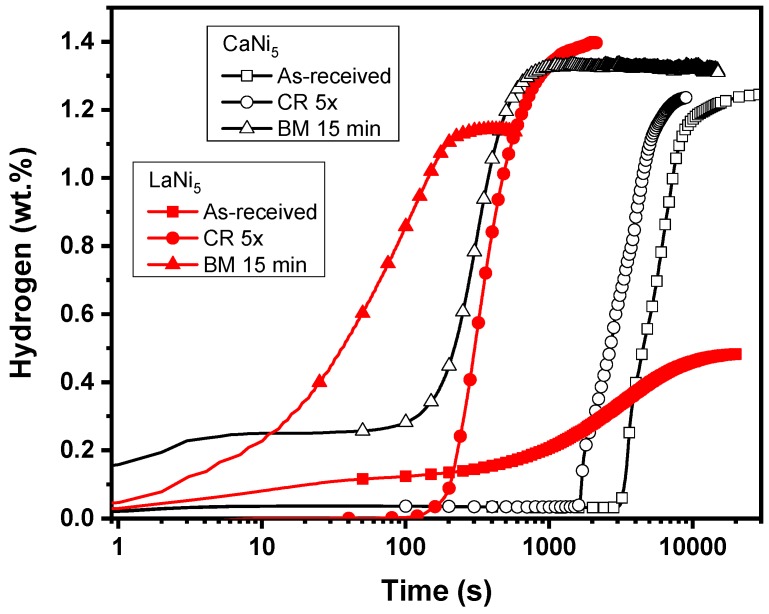
First hydrogenation at 323 K under 1.5 MPa of hydrogen of CaNi_5_ and LaNi_5_ in their as-received, cold rolled and ball milled states. (Adapted from refs. [236,237]).

Figure 22 shows the first hydrogenation of CaNi_5_ and LaNi_5_ in their as-received, cold rolled and ball milled states. Several features can be seen in this figure. First, the as-received powders have a long incubation time with a relatively slow kinetics afterwards. In the case of LaNi_5_, the total capacity is much less than the theoretical one even after waiting for almost 20,000 s (5.5 h). Second, the effect of cold rolling is to reduce the incubation time and increase the kinetic for both alloys but, for the CaNi_5_ alloy, the capacity is also greatly increased. Thirdly, ball milling produced a much faster kinetic and also the initial capacity was higher, both samples readily absorbing a small amount of hydrogen after only a few seconds. However, the CaNi_5_ alloy presented a reduction of capacity. In the assessment of both methods, one has to consider that cold rolling was done in air while ball milling was done under argon. This makes cold rolling much easier and faster to perform than ball milling. In addition, cold rolling could be processed in continuous mode while ball milling is a batch process. Therefore, for commercial applications, cold rolling is probably a better candidate for powder processing than ball milling.

Even though the utilization of cold rolling for metal hydrides is relatively new, in addition to these two examples, many other systems have been investigated [214,217,225,227,239,240,241,242,243]. However, there is still a lack of understanding the exact reason for the effectiveness of cold rolling on the hydrogenation behavior of metal hydride. As cold rolling is a well-known industrial technique, it is worthwhile to continue fundamental research and studying scaling-up problems when this technique is applied to metal hydrides.

## 8. In Situ Characterization of the Mechanochemical Reaction

In Section 4, we saw that pressure and temperature can be monitored in real time during milling (such as in GTM jars manufactured by Fritsch). These measurements can be seen as an in situ characterization of the process, as it allows to detect exothermic events or the steps of gas uptake or release. Such reaction profiles give the researchers an idea about the reaction profile and, in particular, when the milling process should be stopped in order to do an ex-situ characterization of the reaction products. This study of the reaction path is better guided than the blind search along the axis of the reaction time. However, the ex-situ characterization has a number of shortcomings. The first is a problem detecting short living intermediates. Therefore, the intermediate reaction steps in mechanochemical synthesis remain poorly understood by traditional ex-situ characterization of the reaction mixture.

### 8.1. First In Situ Diffraction Studies

A significant step forward in the in situ characterization of the reaction mixture has been made using a combination of mechano-chemical synthesis with synchrotron X-ray powder diffraction [244,245]. The high-energy X-ray beam was sent through thick walled (3.1 mm each wall) plastic jars where a mechanochemical reaction was performed using a shaker mill. One or several stainless-steel balls can be used with little time (or even no time given some optimization) to get their trajectories onto the path of the X-ray beam. The use of high-intensity synchrotron beam allowed reaching time resolution of a few seconds. For example, this setup allowed detecting an intermediate in the mechanochemical synthesis of the metal-organic framework ZIF-8 having a new topology [246]. Already a year later, the first studies using this setup were done on the hydride systems [247]. This method allowed detecting even light hydrides containing weakly scattering elements and has shown a good time resolution. However, the following limits were identified:

- High energies (short wavelengths) are needed to penetrate the thick-walled plastic jars. This limits the use of the setup only to high-energy synchrotron beamlines with λ or the order of ~0.15 Å.
- The use of high-energy X-rays has another drawback: the powder diffraction data are squeezed in a short 2θ-range, with strongly overlapping Bragg peaks, making it difficult to collect high-resolution data.
- The thick-walled plastic jars produce high amorphous background, which needs to be subtracted for presentation purposes when plotting data for weakly scattering samples, such as hydrides.
- The plastic jars have to be machined in a workshop at relatively high price and they do not stand many milling cycles, being especially prone to degradation when using small amounts of solvents in liquid assisted grinding.
- The large sample volume gives rise to broad or even split diffraction peaks coming from the sample sticking on the opposite sides of the milling jar. In part, this problem can be solved by hitting the milling volume by X-ray beam close to the inner wall edge. Thus, the X-ray beam should not go through the middle of the milling jar but close to its edge. This also reduces the probability of hitting the metal balls with the X-ray beam.

**Figure 23:**
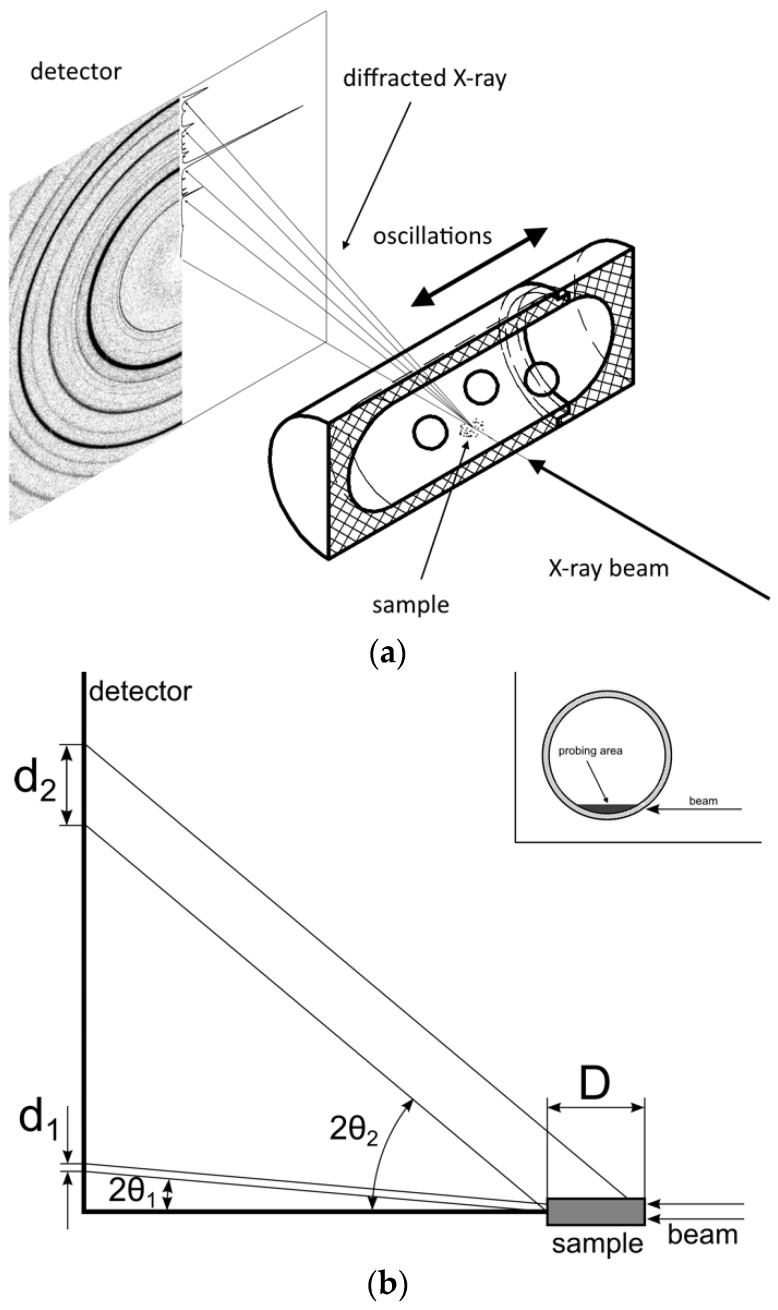
The scheme of in situ ball-milling experiment monitored by powder X-ray diffraction using a shaker mill (**a**). The X-ray beam goes through the bottom of the oscillating plastic jar containing the sample and metal balls. Diffracted X-rays are registered by a 2D detector. The jar is positioned so that the X-ray beam is hitting the sample on the inner wall (on the bottom). The resolution function: The width of the diffraction peaks dependd on the 2θ angle (**b**). D is the sample thickness; and d_1_ and d_2_ are the projections of the sample on the detector at diffraction angles of 2θ_1_ and 2θ_2_, respectively. The Figures are taken from [255].

Indeed, the diffraction on the milling balls add an extra complexity. This can be avoided by finding appropriate shaking frequencies allowing for stable trajectories of the milling balls. In this case, it is possible to probe the space where the milling balls. The sketch of the resulting set up and the illustration of the resolution function are shown in Figure 23.

This technique is becoming increasingly popular in different fields of mechanochemistry [248,249,250,251,252] and was applied to situ Raman spectroscopy [253] as well as in combined Raman/diffraction/thermography studies [254].

### 8.2. Improving the Design of the Milling Jars by 3D Printing

The drawbacks listed above were considered in building the next generation of the milling jars, which had an optimized shape and lower cost, all thanks to the 3D printing technologies becoming widely available in recent years. The 3D-printed polylactic acid (PLA) jars show good mechanical strength; they are also more resistant to solvents compared to polymethyl methacrylate (PMMA). There materials are robust enough to withstand the impacts from the grinding balls; they are amorphous and thus produce no diffraction peaks; and they are quite resistant to solvents allowing for liquid-assisted grinding (LAG).

**Figure 24:**
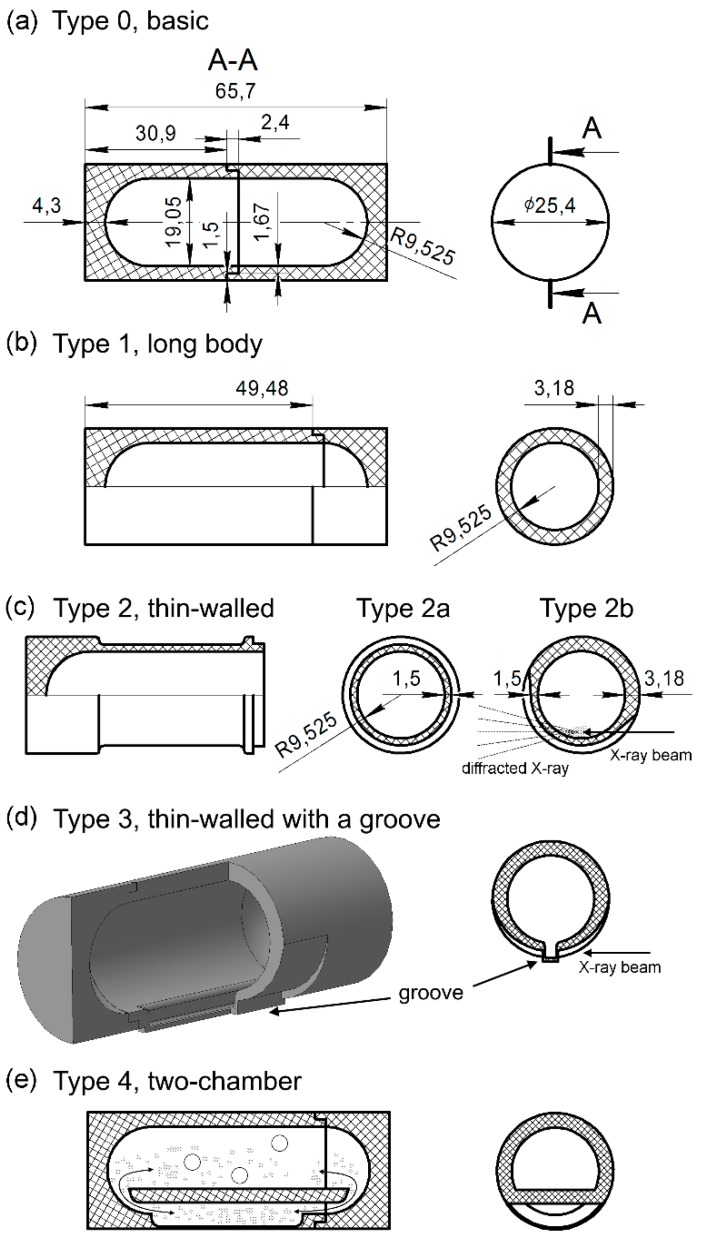
Designs of the 3D printed milling jars: (**a**) Type 0 jar, basic design from Ref. [1]; (**b**) Type 1 jar, with a joint point near the end of the jar; (**c**) Type 2 thin-walled jar; (**d**) Type 3 thin-walled jar with a groove; and (**e**) Type 4 two-chamber jar.

Compared to the first-generation jars described above, a major idea of the improvement here was to reduce the wall thickness of the jar in order to decrease its contribution to the background. In addition, the sample thickness is reduced in order to keep good resolution at high angles. One way to do this is using a design of a jar with two separate chambers: one in which the actual ball-milling reaction occurs, and another in which the powder is characterized by X-ray diffraction. This and other designs are shown in Figure 24.

The modified wall thickness, the use of a thin-walled sampling groove and of a two-chamber design, where the milling and diffraction take place in two communicating volumes, allow for a reduced background/absorption and higher angular resolution, with a perspective for use at lower energy beamlines. Although all the tested jars showed good efficiency, the main drawback of the Type 3 and 4 jars is that they are not suitable for liquid-assisted grinding experiment. In practice, wet powders easily stick inside the narrow parts of the jars, thus the material exposed to X-rays is not taking part in the reaction. On the other hand, the Type 3 thin-walled jars with a groove is seen as the most promising for measurements at low energy beamlines aiming for relatively high angular resolution: the thin-walled groove contains the sample within a small volume, providing both for a low background/absorption and for higher resolution in the reciprocal space. Besides the improved resolution and the background, the big advantage of this approach is the ease of use and low cost: the source files for printing the jars are available as a supporting information in [255].

### 8.3. Going for Ultimate Angular Resolution and an Efficient Sampling

**Figure 25:**
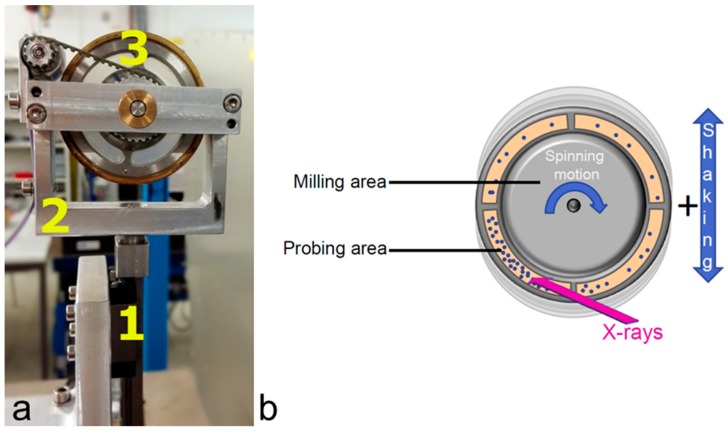
(**a**) Ball mill setup with the stroking device marked in 1, the frame holder in 2 and the jar container in 3; and (**b**) schematic of the jar container and motion principles [256].

Further improvement was achieved using a relatively complicated reaction cell, shown in Figure 25. The innovative part is the grinding container, which follows the vertical grinding motion going up to 50 Hz provided by Pulverisette 23 from Fritsch. The vessel can also rotate continuously on its axis at a rate of 0.1–0.5 Hz. Frequency of the milling and rotation speed of the jar are remotely controlled to synchronize the experiment outside the hutch. A lower energy X-ray beam of λ ~0.7 Å is typically used. In situ ball milling experiments were performed at the X04SA Materials Science (MS) beamline at the Swiss Light Source (SLS), Paul Scherrer Institute. During a typical in situ ball milling experiment, the X-ray beam passes through the probing windows while the jar is vigorously shaking and slowly spinning. Scattered X-rays were detected with a 1D multistrip detector Mythen II or a 2D hybrid pixel array detector Pilatus 6M. This setup is available there to MS beamline users since September 2016.

The particular geometry of the grinding vessel results in PXRD data displaying significantly lower background and much sharper Bragg peaks, which in turn allow more sophisticated analysis of mechanochemical processes. Indeed, the FWHM of the standard LaB_6_ in this setup is about the same as in a 0.8 mm capillary. This main improvement in the peak width would not have been possible if X-rays were passing through the entire jar. The extra spinning motion of the milling jar is also a big improvement in the way of measuring the sample. With the previous setups [244,245,255], powder could be trapped inside the probing area, biasing the true reaction advancement as the same part was probed over time. This cannot occur with the present prototype for neat grinding, although this may be still problematic in the case of liquid-assisted grinding. Moreover, with this recent design, it will likely be easier and more efficient to couple the in-situ diffraction with other analytical techniques.

**Figure 26:**
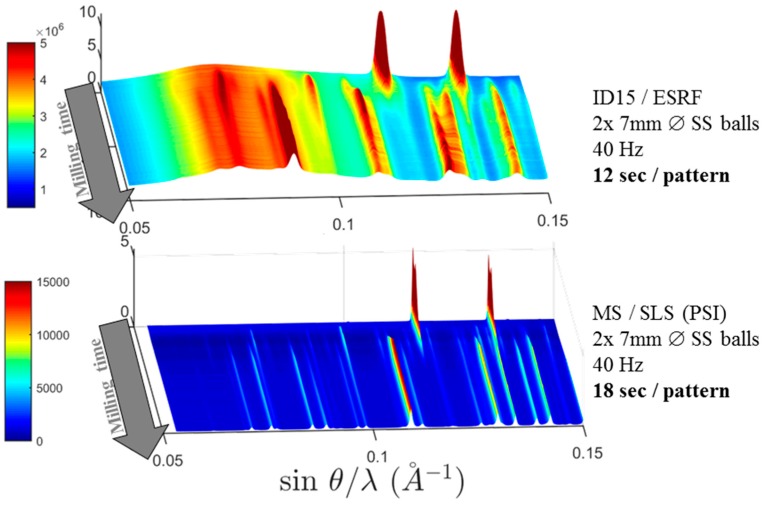
Comparison of XRD patterns collected during the mechanochemical reaction between LiBH_4_ and CsBH_4_, taken at high energy ESRF beamline (former ID15, 90 keV) and at medium energy MS beamline at SLS (16 keV) [256].

With regard to the synthesis of hydrides, the clear advantage of the improved resolution is illustrated in Figure 26, showing the data collected at high energy beamline using the original setup as described in Refs. [244,245] and the most recent design described in Ref. [256].

## 9. Conclusions

Mechanosynthesis is a powerful way to synthesize and modify hydrogen storage materials. In this paper, we show that through mechanosynthesis a wide range of complex hydrides can be synthesized. In fact, mechanosynthesis is sometimes the only way, or at least the most efficient way, to make these compounds. In addition, milling at low temperature (cryomilling) or under hydrogen pressure enables the synthesis of new materials in a relatively easy way. In recent years, new synthesis methods, collectively known as Severe Plastic Deformation (SPD) techniques, have been used to prepare metal hydrides. In this paper, we demonstrate the high efficiency of High-Pressure Torsion (HPT), Surface Mechanical Attrition Treatment (SMAT), and cold rolling to enhance the hydrogen storage properties of metal hydrides. Using these techniques, the effect of grain boundaries, dislocations, stacking faults, and amorphous phases can be studied. Fundamental understanding of the mechanochemistry process can now be better studied thanks to the new technique of in situ X-ray diffraction. These development of new synthesis and characterization techniques are proof that the field of mechanochemistry has a bright future and will continue to bring new and exciting discoveries.

## Figures and Tables

**Table 1:** Metal borohydrides synthesized by mechanochemical methods, reactants used for the synthesis, optimal reactant ratio, formed side products, total milling time (tmt in minutes) and milling speed (revolutions per minute, rpm) used in the synthesis [5].

Compound	Reactants Opt.	Ratio	Side Products	tmt	rpm	Ref.
NaBH_4_	MgH_2_ + Na_2_B_4_O_7_	4:1	MgO, B_2_O_3_	60	2750	[28,59]
KBH_4_	MgH_2_ + KBO_2_	2:1	MgO	120	490	[60]
Sr(BH_4_)Cl	LiBH_4_ + SrCl_2_	1:1	LiCl, Sr(BH_4_)_2_	120	400	[61]
Sr(BH_4_)_2_	LiBH_4_ + SrCl_2_	1:1	LiCl, Sr(BH_4_)Cl	120	400	[61]
LiSc(BH_4_)_4_	LiBH_4_ + ScCl_3_	4:1	LiCl	180	500	[62,63,64]
NaSc(BH_4_)_4_	NaBH_4_ + ScCl_3_	2:1	Na_3_ScCl_6_	120	400	[57]
KSc(BH_4_)_4_	KBH_4_ + ScCl_3_	2:1	K_3_ScCl_6_	120	400	[58]
Y(BH_4_)_3_	LiBH_4_ + YCl_3_	3:1	LiCl	120	200	[36,37]
NaY(BH_4_)_2_Cl_2_	NaBH_4_ + YCl_3_	2:1	Na_3_YCl_6_, Na(BH_4_)_1–x_Cl_x_	120	200	[65]
Mn(BH_4_)_2_	LiBH_4_ + MnCl_2_	2:1	LiCl	350	600	[66]
Mn(BH_4_)_2_	NaBH_4_ + MnCl_2_	2:1	NaCl	350	600	[66]
LiZn_2_(BH_4_)_5_	LiBH_4_ + ZnCl_2_	5:2	LiCl	120	200	[54]
NaZn_2_(BH_4_)_5_	NaBH_4_ + ZnCl_2_	5:2	Na_2_ZnCl_4_, NaCl	120	200	[54]
NaZn(BH_4_)_3_	NaBH_4_ + ZnCl_2_	3:1	Na_2_ZnCl_4_, NaCl	120	200	[54]
KZn(BH_4_)Cl_2_	KBH_4_ + ZnCl_2_	1:1	-	120	200	[51]
Cd(BH_4_)_2_	LiBH_4_ + CdCl_2_	2:1	LiCl	30	200	[67]
Cd(BH_4_)_2_	NaBH_4_ + CdCl_2_	14:9	NaCl, Na_6_CdCl_8_	30	200	[67]
KCd(BH_4_)_3_	KBH_4_ + CdCl_2_	1:1	KCdCl_3_, K_2_Cd(BH_4_)_4_, Cd(BH_4_)_2_	20	200	[67]
K_2_Cd(BH_4_)_4_	KBH_4_ + CdCl_2_	4:3	KCdCl_3_	20	200	[67]
Li_4_Al_3_(BH_4_)_13_	LiBH_4_ + AlCl_3_	13:3	LiCl	300	500	[68]
Li(BH_4_)_0.9_Cl_0.1_	LiBH_4_ + LiCl	-	-	120	200	[69,70]
Li(BH_4_)_0.47_Br_0.53_	LiBH_4_ + LiBr	-	-	120	200	[71]
Li(BH_4_)_0.3_I_0.7_	LiBH_4_ + LiI	-	-	120	200	[72]
LiBH_4_-NaBH_4_	LiBH_4_+NaBH_4_	1:1	LiNaBH_4_	300	175	[73,74]
LiBH_4_-NaBH_4_-Ni	LiBH_4_+NaBH_4_ +Ni	-	Ni_4_B_3_, Ni_2_B, Ni_3_B	300	175	[74]
Na(BH_4_)_0.9_Cl_0.1_	NaBH_4_ + NaCl	-	-	120	200	[75]
Ca(BH_4_)_1.6_I_0.4_	Ca(BH_4_)_2_ + CaI_2_	-	-	120	250	[76]
LiLa(BH_4_)_3_Cl	La(BH_4_)_3_ + LiCl	1:1	unknown phase	120	350	[77]
LiLa(BH_4_)_3_Br	La(BH_4_)_3_ + LiBr	1:1	unknown phase	120	350	[77]
LiLa(BH_4_)_3_I	La(BH_4_)_3_ + LiI	1:1	unknown phase	120	350	[77]
NaCe(BH_4_)_4_	NaBH_4_ + Ce(BH_4_)_3_	1:1	β-Ce(BH_4_)_3_, unknown phase	120	350	[78]
NaPr(BH_4_)_4_	NaBH_4_ + Pr(BH_4_)_3_	1:1	unknown phase	120	350	[78]
NaEr(BH_4_)_4_	NaBH_4_ + Er(BH_4_)_3_	1:1	-	120	350	[78]
Na(BH_4_)_1-x_Br_x_	NaBH_4_ + NaBr	1:1	-	360	200	[79]
KEr(BH_4_)_4_	KBH_4_ + Er(BH_4_)_3_	1:1	-	60	-	[80]

**Table 2:** Formation of metastable phases in various Ti-based and Mg-based systems by HPT processing.

System	Metastable Phase(s)	Ref.
Ti-V	bcc	[166]
Ti-Nb	bcc	[172]
Mg-Al	amorphous	[174]
Mg-Zn	amorphous	[174]
Mg-Sn	Hcp	[174]
Mg-Ti	bcc, hcp, fcc	[173]
Mg-Zr	bcc, hcp, fcc	[175]
Mg-V	bcc	[169]
Mg-V-Ni	bcc	[170]
Mg-V-Pd	bcc, CsCl-type	[170]
Mg-V-Sn	CsCl-type	[170]
Mg-V-Cr	bcc	[169]
Mg-Ni-Sn	amorphous	[170]
Mg-Ni-Pd	CsCl-type	[168]

## References

[1] Benjamin J.S. (1976). Mechanical Alloying. Sci. Am..

[2] Benjamin J.S., Volin T.E. (1974). The mechanism of mechanical alloying. Met. Mater. Trans. A.

[3] Suryanarayana C. (2001). Mechanical alloying and milling. Prog. Mater. Sci..

[4] Richter B., Grinderslev J.B., Møller K.T., Paskevicius M., Jensen T.R. (2018). From Metal Hydrides to Metal Borohydrides. Inorg. Chem..

[5] Huot J., Ravnsbæk D., Zhang J., Cuevas F., Latroche M., Jensen T.R. (2013). Mechanochemical synthesis of hydrogen storage materials. Prog. Mater. Sci..

[6] Balema V.P., Wiench J.W., Pruski M., Pecharsky V.K. (2002). Mechanically Induced Solid-State Generation of Phosphorus Ylides and the Solvent-Free Wittig Reaction. J. Am. Chem. Soc..

[7] Hagemann H., Cerny R. (2010). Synthetic approaches to inorganic borohydrides. Dalton Trans..

[8] Beyer M.K., Clausen-Schaumann H. (2005). Mechanochemistry: The Mechanical Activation of Covalent Bonds. Chem. Rev..

[9] Bösenberg U., Doppiu S., Mosegaard L., Barkhordarian G., Eigen N., Borgschulte A., Jensen T.R., Cerenius Y., Gutfleisch O., Klassen T. (2007). Hydrogen sorption properties of MgH_2_–LiBH_4_ composites. Acta Mater..

[10] Fichtner M. (2005). Nanotechnological Aspects in Materials for Hydrogen Storage. Adv. Eng. Mater..

[11] Blanchard D., Brinks H.W., Hauback B.C. (2006). Isothermal decomposition of LiAlD_4_. J. Alloys Compd..

[12] Callini E., Atakli Z.Ö.K., Hauback B.C., Orimo S.-I., Jensen C., Dornheim M., Grant D., Cho Y.W., Chen P., Hjörvarsson B. (2016). Complex and liquid hydrides for energy storage. Appl. Phys. A.

[13] Møller K.T., Jensen T.R., Akiba E., Li H.-W. (2017). Hydrogen—A sustainable energy carrier. Prog. Nat. Sci..

[14] Møller K.T., Sheppard D., Ravnsbæk D.B., Buckley C.E., Etsuo A., Hai-Wen L., Jensen T.R. (2017). Complex Metal Hydrides for Hydrogen. Thermal and Electrochemical Energy Storage. Energies.

[15] Callini E., Aguey-Zinsou K.-F., Ahuja R., Ares J.R., Bals S., Biliškov N., Chakraborty S., Charalambopoulou G., Chaudhary A.-L., Cuevas F. (2016). Nanostructured materials for solid-state hydrogen storage: A review of the achievement of COST Action MP1103. Int. J. Hydrog. Energy.

[16] Crivello J.-C., Dam B., Denys R.V., Dornheim M., Grant D.M., Huot J., Jensen T.R., De Jongh P., Latroche M., Milanese C. (2016). Review of magnesium hydride-based materials: Development and optimisation. Appl. Phys. A.

[17] Crivello J.-C., Denys R.V., Dornheim M., Felderhoff M., Grant D.M., Huot J., Jensen T.R., De Jongh P., Latroche M., Walker G.S. (2016). Mg-based compounds for hydrogen and energy storage. Appl. Phys. A.

[18] Yartys V.A., Lototskyy M.V., Akiba E., Albert R., Antonov V.E., Ares J.R., Baricco M., Bourgeois N., Buckley C.E., Bellosta von Colbe J.M. (2019). Magnesium based materials for hydrogen based energy storage: Past, present and future. Int. J. Hydrog. Energy.

[19] Milanese C., Jensen T., Hauback B., Pistidda C., Dornheim M., Yang H., Lombardo L., Zuettel A., Filinchuk Y., Ngene P. (2019). Complex hydrides for energy storage. Int. J. Hydrog. Energy.

[20] Von Colbe J.B., Ares J.-R., Barale J., Baricco M., Buckley C., Capurso G., Gallandat N., Grant D.M., Guzik M.N., Jacob I. (2019). Application of hydrides in hydrogen storage and compression: Achievements, outlook and perspectives. Int. J. Hydrog. Energy.

[21] Manickam K., Mistry P., Walker G., Grant D., Buckley C.E., Humphries T.D., Paskevicius M., Jensen T., Albert R., Peinecke K. (2019). Future perspectives of thermal energy storage with metal hydrides. Int. J. Hydrog. Energy.

[22] Latroche M., Blanchard D., Cuevas F., El Kharbachi A., Hauback B.C., Jensen T.R., De Jongh P.E., Kim S., Nazer N.S., Ngene P. (2019). Full-cell hydride-based solid-state Li batteries for energy storage. Int. J. Hydrog. Energy.

[23] Fritsch, Product Information, Planetary Mills Classic Line. http://www.fritsch.de/uploads/media/e_Planetary_Mills_classic_line.pdf.

[24] Schilz J. (1998). Internal Kinematics of Tumbler and Planetary Ball Mills: A Mathematical Model for the Parameter Setting. Mater. Trans. JIM.

[25] Burgio N., Iasonna A., Magini M., Martelli S., Padella F. (1991). Mechanical alloying of the Fe−Zr system. Correlation between input energy and end products. Il Nuovo Cim. D.

[26] Murty B., Rao M.M., Ranganathan S. (1995). Milling maps and amorphization during mechanical alloying. Acta Met. Mater..

[27] Orimo S.-I., Nakamori Y., Eliseo J.R., Zuettel A., Jensen C.M., Orimo S. (2007). Complex Hydrides for Hydrogen Storage. Chemin.

[28] Schlesinger H.I., Brown H.C., Finholt A.E. (1953). The Preparation of Sodium Borohydride by the High Temperature Reaction of Sodium Hydride with Borate Esters1. J. Am. Chem. Soc..

[29] Černý R., Schouwink P. (2015). The crystal chemistry of inorganic metal boro-hydrides and their relation to metal oxides. Acta Crystallogr. Sect. B Struct. Sci. Cryst. Eng. Mater..

[30] Paskevicius M., Jepsen L.H., Schouwink P., Černý R., Ravnsbæk D.B., Filinchuk Y., Dornheim M., Besenbacher F., Jensen T.R. (2017). Metal borohydrides and derivatives—Synthesis, structure and properties. Chem. Soc. Rev..

[31] Frommen C., Sørby M.H., Heere M., Humphries T.D., Olsen J.E., Hauback B.C. (2017). Rare Earth Borohydrides—Crystal Structures and Thermal Properties. Energies.

[32] Sato T., Miwa K., Nakamori Y., Ohoyama K., Li H.-W., Noritake T., Aoki M., Towata S.-I., Orimo S.-I. (2008). Experimental and computational studies on solvent-free rare-earth metal borohydridesR(BH_4_)_3_(R = Y, Dy, and Gd). Phys. Rev. B.

[33] Olsen J.E., Frommen C., Sørby M., Hauback B.C. (2013). Crystal structures and properties of solvent-free LiYb(BH_4_)_4−x_Cl_x_, Yb(BH_4_)_3_ and Yb(BH_4_)_2−x_Cl_x_. RSC Adv..

[34] Frommen C., Sørby M., Ravindran P., Vajeeston P., Fjellvåg H., Hauback B., Sørby M. (2011). Synthesis, Crystal Structure, and Thermal Properties of the First Mixed-Metal and Anion-Substituted Rare Earth Borohydride LiCe(BH_4_)_3_Cl. J. Phys. Chem. C.

[35] Jaron T., Grochala W. (2010). Y(BH_4_)_3_-an old-new ternary hydrogen store aka learning from a multitude of failures. Dalton Trans..

[36] Frommen C., Aliouane N., Deledda S., Fonneløp J.E., Grove H., Lieutenant K., Llamas-Jansa I., Sartori S., Sørby M.H., Hauback B.C. (2010). Crystal structure, polymorphism, and thermal properties of yttrium borohydride Y(BH_4_)_3_. J. Alloys Compd..

[37] Ravnsbæk D.B., Filinchuk Y., Černyý R., Ley M.B., Haase D., Jakobsen H.J., Skibsted J., Jensen T.R. (2010). Thermal Polymorphism and Decomposition of Y(BH_4_)_3_. Inorg. Chem..

[38] Ley M.B., Jepsen L.H., Lee Y.-S., Cho Y.W., Von Colbe J.M.B., Dornheim M., Rokni M., Jensen J.O., Sloth M., Filinchuk Y. (2014). Complex hydrides for hydrogen storage—New perspectives. Mater. Today.

[39] Yan Y., Li H.-W., Sato T., Umeda N., Miwa K., Towata S.-I., Orimo S.-I. (2009). Dehydriding and rehydriding properties of yttrium borohydride Y(BH_4_)_3_ prepared by liquid-phase synthesis. Int. J. Hydrog. Energy.

[40] Koźmiński W., Grochala W., Jaroń T. (2011). Phase transition induced improvement in H_2_ desorption kinetics: The case of the high-temperature form of Y(BH_4_)_3_. Phys. Chem. Chem. Phys..

[41] Gennari F., Esquivel M. (2009). Synthesis and dehydriding process of crystalline Ce(BH_4_)_3_. J. Alloys Compd..

[42] Li H.-W., Yan Y., Orimo S.-I., Züttel A., Jensen C.M. (2011). Recent Progress in Metal Borohydrides for Hydrogen Storage. Energies.

[43] Zhang B.J., Liu B.H., Li Z.P. (2011). Destabilization of LiBH_4_ by (Ce, La)(Cl, F)_3_ for hydrogen storage. J. Alloys Compd..

[44] Ley M.B., Boulineau S., Janot R., Filinchuk Y., Jensen T.R. (2012). New Li Ion Conductors and Solid State Hydrogen Storage Materials: LiM(BH_4_)_3_Cl, M = La, Gd. J. Phys. Chem. C.

[45] Skripov A.V., Soloninin A.V., Ley M.B., Jensen T.R., Filinchuk Y. (2013). Nuclear Magnetic Resonance Studies of BH 4 Reorientations and Li Diffusion in LiLa(BH_4_)_3_ Cl. J. Phys. Chem. C.

[46] Gennari F., Albanesi L.F., Puszkiel J., Larochette P.A. (2011). Reversible hydrogen storage from 6LiBH_4_–MCl_3_ (M = Ce, Gd) composites by in-situ formation of MH_2_. Int. J. Hydrog. Energy.

[47] Heere M., Gharibdoust S.H.P., Frommen C., Humphries T.D., Ley M.B., Sørby M.H., Jensen T.R., Hauback B.C. (2016). The influence of LiH on the rehydrogenation behavior of halide free rare earth (RE) borohydrides (RE = Pr, Er). Phys. Chem. Chem. Phys..

[48] Schouwink P., Ley M.B., Jensen T.R., Černý R. (2014). Borohydrides: From sheet to framework topologies. Dalton Trans..

[49] Grinderslev J.B., Møller K.T., Bremholm M., Jensen T.R. (2019). Trends in Synthesis, Crystal Structure, and Thermal and Magnetic Properties of Rare-Earth Metal Borohydrides. Inorg. Chem..

[50] Lee Y.-S., Shim J.-H., Cho Y.W. (2010). Polymorphism and Thermodynamics of Y(BH_4_)_3_ from First Principles. J. Phys. Chem. C.

[51] Ravnsbaek D.B., Sørensen L.H., Filinchuk Y., Reed D., Book D., Jakobsen H.J., Besenbacher F., Skibsted J., Jensen T.R., Ravnsbæk D.B. (2010). Mixed-Anion and Mixed-Cation Borohydride KZn(BH_4_)Cl_2_: Synthesis, Structure and Thermal Decomposition. Eur. J. Inorg. Chem..

[52] Ley M.B., Ravnsbæk D.B., Filinchuk Y., Janot R., Cho Y.W., Lee Y.-S., Skibsted J., Jensen T.R. (2012). LiCe(BH_4_)_3_Cl, a New Lithium-Ion Conductor and Hydrogen Storage Material with Isolated Tetranuclear Anionic Clusters. Chem. Mater..

[53] Frommen C., Heere M., Riktor M.D., Sørby M.H., Hauback B.C. (2015). Hydrogen storage properties of rare earth (RE) borohydrides (RE = La, Er) in composite mixtures with LiBH_4_ and LiH. J. Alloys Compd..

[54] Ravnsbaek D., Filinchuk Y., Cerenius Y., Jakobsen H.J., Besenbacher F., Skibsted J., Jensen T.R. (2009). A Series of Mixed-Metal Borohydrides. Angew. Chem. Int. Ed..

[55] Cerný R., Chul Kim K., Penin N., Da’nna V., Hagemann H., Sholl D.S. (2010). AZn_2_(BH_4_)_5_(A = Li, Na) and NaZn(BH_4_)_3_: Structural studies. J. Phys. Chem. C.

[56] Solinas I., Lutz H.D. (1995). Nonceramic preparation techniques for ternary halides AB_2 × 4_ with A = Mg, Mn, Zn B = Li, Na X = Cl, Br-1. J. Solid State Chem..

[57] Černý R., Severa G., Ravnsbæk D.B., Filinchuk Y., D’Anna V., Hagemann H., Haase D., Jensen C.M., Jensen T.R. (2010). NaSc(BH_4_)_4_: A novel scandium-based borohydride. J. Phys. Chem. C.

[58] Černyý R., Ravnsbæk D.B., Severa G., Filinchuk Y., Anna V.D., Hagemann H., Haase D., Skibsted J., Jensen C.M., Jensen T.R. (2010). Structure and Characterization of KSc(BH_4_)_4_. J. Phys. Chem. C.

[59] Li Z., Morigazaki N., Liu B., Suda S. (2003). Preparation of sodium borohydride by the reaction of MgH_2_ with dehydrated borax through ball milling at room temperature. J. Alloys Compd..

[60] Li Z.P., Liu B.H., Morigazaki N., Suda S. (2003). Preparation of potassium borohydride by a mechanico-chemical reaction of saline hydrides with dehydrated borate through ball milling. J. Alloys Compd..

[61] Ravnsbæk D.B., Nickels E.A., Cerny R., Olesen C.H., David W.I.F., Edwards P.P., Filinchuk Y., Jensen T.R. (2013). Novel Alkali Earth Borohydride Sr(BH_4_)_2_ and Borohydride-Chloride Sr(BH_4_)Cl. Inorg. Chem..

[62] Hagemann H., Longhini M., Kaminski J.W., Wesolowski T.A., Černyý R., Penin N., Sørby M.H., Hauback B.C., Severa G., Jensen C.M. (2008). LiSc(BH_4_)_4_: A Novel Salt of Li+ and Discrete Sc(BH_4_)_4_−Complex Anions. J. Phys. Chem. A.

[63] Hwang S.-J., Bowman R.C., Reiter J.W., Rijssenbeek J., Soloveichik G.L., Zhao J.-C., Kabbour H., Ahn C.C., Rijssenbeek J. (2008). NMR Confirmation for Formation of [B_12_H_12_]^2−^ Complexes during Hydrogen Desorption from Metal Borohydrides. J. Phys. Chem. C.

[64] Kim C., Hwang S.-J., Bowman R.C., Reiter J.W., Zan J.A., Kulleck J.G., Kabbour H., Majzoub E.H., Ozolins V. (2009). LiSc(BH_4_)_4_ as a Hydrogen Storage Material: Multinuclear High-Resolution Solid-State NMR and First-Principles Density Functional Theory Studies. J. Phys. Chem. C.

[65] Ravnsbæk D.B., Ley M.B., Lee Y.-S., Hagemann H., D’Anna V., Cho Y.W., Filinchuk Y., Jensen T.R. (2012). A mixed-cation mixed-anion borohydride NaY(BH_4_)_2_Cl_2_. Int. J. Hydrog. Energy.

[66] Cerny R., Penin N., D’Anna V., Hagemann H., Durand E., Růžička J. (2011). MgxMn(1−x)(BH_4_)_2_ (x = 0–0.8), a cation solid solution in a bimetallic borohydride. Acta Mater..

[67] Ravnsbaek D.B., Sørensen L.H., Filinchuk Y., Besenbacher F., Jensen T.R., Ravnsbæk D.B. (2012). Screening of Metal Borohydrides by Mechanochemistry and Diffraction. Angew. Chem..

[68] Lindemann I., Ferrer R.D., Dunsch L., Filinchuk Y., Černý R., Hagemann H., D’Anna V., Daku L.M.L., Schultz L., Gutfleisch O. (2010). Al_3_Li_4_(BH_4_)_13_: A Complex Double-Cation Borohydride with a New Structure. Chem. A Eur. J..

[69] Mosegaard L., Møller B., Jørgensen J.-E., Filinchuk Y., Cerenius Y., Hanson J.C., DiMasi E., Besenbacher F., Jensen T.R. (2008). Reactivity of LiBH_4_: In Situ Synchrotron Radiation Powder X-ray Diffraction Study. J. Phys. Chem. C.

[70] Arnbjerg L.M., Ravnsbæk D.B., Filinchuk Y., Vang R.T., Cerenius Y., Besenbacher F., Jørgensen J.-E., Jakobsen H.J., Jensen T.R. (2009). Structure and Dynamics for LiBH_4_−LiCl Solid Solutions. Chem. Mater..

[71] Rude L., Zavorotynska O., Arnbjerg L., Ravnsbæk D.B., Malmkjær R., Grove H., Hauback B., Baricco M., Filinchuk Y., Besenbacher F. (2011). Bromide substitution in lithium borohydride, LiBH_4_–LiBr. Int. J. Hydrog. Energy.

[72] Rude L.H., Groppo E., Arnbjerg L.M., Ravnsbæk D.B., Malmkjær R.A., Filinchuk Y., Baricco M., Besenbacher F., Jensen T.R. (2011). Iodide substitution in lithium borohydride, LiBH_4_–LiI. J. Alloys Compd..

[73] Liu Y., Reed D., Paterakis C., Vasquez L.C., Baricco M., Book D. (2017). Study of the decomposition of a 0.62LiBH_4_–0.38NaBH_4_ mixture. Int. J. Hydrog. Energy.

[74] Liu Y., Heere M., Vasquez L.C., Paterakis C., Sørby M.H., Hauback B.C., Book D. (2018). Dehydrogenation and rehydrogenation of a 0.62LiBH_4_-0.38NaBH_4_ mixture with nano-sized Ni. Int. J. Hydrog. Energy.

[75] Ravnsbæk D.B., Rude L.H., Jensen T.R. (2011). Chloride substitution in sodium borohydride. J. Solid State Chem..

[76] Rude L.H., Filinchuk Y., Sørby M.H., Hauback B.C., Besenbacher F., Jensen T.R. (2011). Anion Substitution in Ca(BH_4_)_2_−CaI_2_: Synthesis, Structure and Stability of Three New Compounds. J. Phys. Chem. C.

[77] GharibDoust S., Brighi M., Sadikin Y., Ravnsbæk D.B., Černý R., Jensen T., Skibsted J. (2017). Synthesis, Structure and Li Ion Conductivity of LiLa(BH_4_)_3_X, X = Cl, Br, I. J. Phys. Chem. C.

[78] Gharibdoust S.P., Ravnsbæk D.B., Černý R., Jensen T.R. (2017). Synthesis, structure and properties of bimetallic sodium rare-earth (RE) borohydrides, NaRE(BH_4_)_4_, RE = Ce, Pr, Er or Gd. Dalton Trans..

[79] Paterakis C., Guo S., Heere M., Liu Y., Contreras L.F., Sørby M.H., Hauback B.C., Reed D., Book D. (2017). Study of the NaBH_4_–NaBr system and the behaviour of its low temperature phase transition. Int. J. Hydrog. Energy.

[80] Heere M., Gharibdoust S.P., Sørby M.H., Frommen C., Jensen T.R., Hauback B.C. (2017). In situ investigations of bimetallic potassium erbium borohydride. Int. J. Hydrog. Energy.

[81] Reilly J.J., Wiswall R.H. (1967). The reaction of hydrogen with alloys of magnesium and copper. Inorg. Chem..

[82] Vajo J.J., Skeith S.L., Mertens F. (2005). Reversible Storage of Hydrogen in Destabilized LiBH_4_. J. Phys. Chem. B.

[83] Vajo J.J., Mertens F., Ahn C.C., Bowman R.C.J., Fultz B. (2004). Altering Hydrogen Storage Properties by Hydride Destabilization Through Alloy Formation: LiH and MgH_2_ Destabilized with Si. Chemin.

[84] Barkhordarian G., Klassen T., Dornheim M., Bormann R. (2007). Unexpected kinetic effect of MgB2 in reactive hydride composites containing complex borohydrides. J. Alloys Compd..

[85] Barkhordarian G., Klassen T., Bormann R. (2003). Fast hydrogen sorption kinetics of nanocrystalline Mg using Nb_2_O_5_ as catalyst. Scr. Mater..

[86] Barkhordarian G., Klassen T., Bormann R. (2006). Composite Material Storing Hydrogen, and Device for the Reversible Storage of Hydrogen. International Patent Application.

[87] Dornheim M., Doppiu S., Barkhordarian G., Boesenberg U., Klassen T., Gutfleisch O., Bormann R. (2007). Hydrogen storage in magnesium-based hydrides and hydride composites. Scr. Mater..

[88] Garroni S., Pistidda C., Brunelli M., Vaughan G.B.M., Surinach S., Baro M.D. (2009). Hydrogen desorption mechanism of 2NaBH_4_ + MgH_2_ composite prepared by high-energy ball milling. Scr. Mater..

[89] Mao J., Yu X., Guo Z., Liu H.K., Wu Z., Ni J. (2009). Enhanced hydrogen storage performances of NaBH_4_–MgH_2_ system. J. Alloys Compd..

[90] Pistidda C., Napolitano E., Pottmaier D., Dornheim M., Klassen T., Baricco M., Enzo S. (2013). Structural study of a new B-rich phase obtained by partial hydrogenation of 2NaH+ MgB_2_. Int. J. Hydrog. Energy.

[91] Pistidda C., Garroni S., Minella C.B., Dolci F., Jensen T.R., Nolis P., Bösenberg U., Cerenius Y., Lohstroh W., Fichtner M. (2010). Pressure Effect on the 2NaH + MgB_2_ Hydrogen Absorption Reaction. J. Phys. Chem. C.

[92] Heere M., Sørby M.H., Pistidda C., Dornheim M., Hauback B.C. (2016). Milling time effect of Reactive Hydride Composites of NaF NaH MgB_2_ investigated by in situ powder diffraction. Int. J. Hydrog. Energy.

[93] Kim J.W., Shim J.-H., Ahn J.-P., Cho Y.W., Kim J.-H., Oh K.H. (2008). Mechanochemical synthesis and characterization of TiB_2_ and VB_2_ nanopowders. Mater. Lett..

[94] Olsen J.E., Sørby M.H., Hauback B.C. (2011). Chloride-substitution in sodium borohydride. J. Alloys Compd..

[95] Llamas-Jansa I., Aliouane N., Deledda S., Fonneløp J.E., Frommen C., Humphries T., Lieutenant K., Sartori S., Sørby M.H., Hauback B.C. (2012). Chloride substitution induced by mechano-chemical reactions between NaBH_4_ and transition metal chlorides. J. Alloys Compd..

[96] Nakamori Y., Li H., Miwa K., Towata S.-I., Orimo S.-I. (2006). Syntheses and Hydrogen Desorption Properties of Metal-Borohydrides M(BH_4_)n (M = Mg, Sc, Zr, Ti, and Zn; n = 2–4) as Advanced Hydrogen Storage Materials. Mater. Trans..

[97] Nakamori Y., Li H.-W., Kikuchi K., Aoki M., Miwa K., Towata S., Orimo S.-I. (2007). Thermodynamical stabilities of metal-borohydrides. J. Alloys Compd..

[98] Yang C.-H., Tsai W.-T., Chang J.-K. (2011). Hydrogen desorption behavior of vanadium borohydride synthesized by modified mechano-chemical process. Int. J. Hydrog. Energy.

[99] Llamas-Jansa I., Aliouane N., Deledda S., Fonneløp J.E., Frommen C., Lieutenant K., Sartori S., Sørby M.H., Hauback B.C. (2011). Mechano-chemical reactions in LiBH_4_ + VCln (n = 2 and 3) mixtures. J. Alloys Compd..

[100] Korablov D., Ravnsbæk D.B., Ban V., Filinchuk Y., Besenbacher F., Jensen T.R. (2013). Investigation of MBH_4_–VCl_2_, M = Li, Na or K. Int. J. Hydrog. Energy.

[101] Jeon E., Cho Y. (2006). Mechanochemical synthesis and thermal decomposition of zinc borohydride. J. Alloys Compd..

[102] James B., Wallbridge M. (1970). Metal tetrahydroborates. Prog. Inorg. Chem..

[103] Ley M.B., Paskevicius M., Schouwink P., Richter B., Sheppard D.A., Buckley C., Jensen T.R. (2014). Novel solvates M(BH_4_)_3_S(CH_3_)_2_ and properties of halide-free M(BH_4_)_3_(M = Y or Gd). Dalton Trans..

[104] Ley M.B., Frommen C., Munroe K.T., Hauback B.C., Humphries T.D., Jensen T.R. (2015). Crystal structure and in situ decomposition of Eu(BH_4_)_2_ and Sm(BH_4_)_2_. J. Mater. Chem. A.

[105] Visseaux M., Bonnet F. (2011). Borohydride complexes of rare earths, and their applications in various organic transformations. Coord. Chem. Rev..

[106] Ley M.B., Jørgensen M., Černý R., Filinchuk Y., Jensen T.R. (2016). From M(BH_4_)_3_ (M = La, Ce) Borohydride Frameworks to Controllable Synthesis of Porous Hydrides and Ion Conductors. Inorg. Chem..

[107] Olsen J.E., Frommen C., Jensen T.R., Riktor M.D., Sørby M.H., Hauback B.C. (2014). Structure and thermal properties of composites with RE-borohydrides (RE = La, Ce, Pr, Nd, Sm, Eu, Gd, Tb, Er, Yb or Lu) and LiBH_4_. RSC Adv..

[108] Gharibdoust S.P., Heere M., Sørby M.H., Ley M.B., Ravnsbæk D.B., Hauback B.C., Černý R., Jensen T.R. (2016). Synthesis, structure and properties of new bimetallic sodium and potassium lanthanum borohydrides. Dalton Trans..

[109] Gharibdoust S.P., Heere M., Nervi C., Sørby M.H., Hauback B.C., Jensen T.R.R. (2018). Synthesis, structure, and polymorphic transitions of praseodymium(iii) and neodymium(iii) borohydride, Pr(BH_4_)_3_ and Nd(BH_4_)_3_. Dalton Trans..

[110] Gennari F. (2013). Mechanochemical synthesis of erbium borohydride: Polymorphism, thermal decomposition and hydrogen storage. J. Alloys Compd..

[111] Heere M., Gharibdoust S.H.P., Brighi M., Frommen C., Sørby M.H., Černý R., Jensen T.R., Hauback B.C. (2017). Hydrogen Sorption in Erbium Borohydride Composite Mixtures with LiBH_4_ and/or LiH. Inorganics.

[112] West A.R. (2014). Solid State Chemistry and Its Applications.

[113] Riktor M.D., Deledda S., Herrich M., Gutfleisch O., Fjellvåg H., Hauback B.C. (2009). Hydride formation in ball-milled and cryomilled Mg–Fe powder mixtures. Mater. Sci. Eng. B.

[114] Floriano R., Deledda S., Hauback B., Leiva D., Botta W., Botta W. (2017). Iron and niobium based additives in magnesium hydride: Microstructure and hydrogen storage properties. Int. J. Hydrog. Energy.

[115] Ravnsbæk D.B., Frommen C., Reed D., Filinchuk Y., Sørby M., Hauback B.C., Jakobsen H.J., Book D., Besenbacher F., Skibsted J. (2011). Structural studies of lithium zinc borohydride by neutron powder diffraction, Raman and NMR spectroscopy. J. Alloys Compd..

[116] Brower F.M., Matzek N.E., Reigler P.F., Rinn H.W., Roberts C.B., Schmidt D.L., Snover J.A., Terada K. (1976). Preparation and properties of aluminum hydride. J. Am. Chem. Soc..

[117] Brinks H.W., Istad-Lem A., Hauback B.C. (2006). Mechanochemical synthesis and crystal structure of alpha’-AlD3 and alpha-AlD3. J. Phys. Chem. B.

[118] Brinks H., Langley W., Jensen C., Graetz J., Reilly J., Hauback B. (2007). Synthesis and crystal structure of β-AlD3. J. Alloys Compd..

[119] Brinks H.W., Brown C., Jensen C.M., Graetz J., Reilly J.J., Hauback B.C. (2007). The crystal structure of **γ**-AlD3. J. Alloys Compd..

[120] Yartys V.A., Denys R.V., Maehlen J.P., Frommen C., Fichtner M., Bulychev B.M., Emerich H. (2007). Double-Bridge Bonding of Aluminium and Hydrogen in the Crystal Structure of γ-AlH_3_. Inorg. Chem..

[121] Hauback B.C. (2008). Structures of aluminium-based light weight hydrides. Z. Krist..

[122] Graetz J., Hauback B.C. (2013). Recent developments in aluminum-based hydrides for hydrogen storage. MRS Bull..

[123] Grove H., Sørby M.H., Brinks H.W., Hauback B.C. (2007). In situ synchrotron powder X-ray diffraction studies of the thermal decomposition of **β**- and **γ**-AlD3. J. Phys. Chem. C.

[124] Maehlen J., Yartys V., Denys R., Fichtner M., Frommen C., Bulychev B., Pattison P., Emerich H., Filinchuk Y., Chernyshov D. (2007). Thermal decomposition of AlH_3_ studied by in situ synchrotron X-ray diffraction and thermal desorption spectroscopy. J. Alloys Compd..

[125] Sartori S., Opalka S.M., Løvvik O.M., Guzik M.N., Tang X., Hauback B.C. (2008). Experimental studies of **α**-AlD3 and **α**’-AlD3 versus first-principles modelling of the alane isomorphs. J. Mater. Chem..

[126] Sartori S., Istad-Lem A., Brinks H.W., Hauback B.C. (2009). Mechanochemical synthesis of alane. International J. Hydrog. Energy.

[127] Fonneløp J.E., Sartori S., Sørby M.H., Hauback B.C. (2012). Polymorphic composition of alane after cryomilling with fluorides. J. Alloys Compd..

[128] Fonneløp J.E., Corno M., Grove H., Pinatel E., Sørby M.H., Ugliengo P., Baricco M., Hauback B.C. (2011). Experimental and computational investigations on the AlH_3_/AlF_3_ system. J. Alloys Compd..

[129] Brinks H.W., Fossdal A., Hauback B.C. (2008). Adjustment of the stability of complex hydrides through anion substitution. J. Phys. Chem. C.

[130] Zhang J., Li Z., Cuevas F., Latroche M. (2014). Phase Stabilities in the Mg–Si–H System Tuned by Mechanochemistry. J. Phys. Chem. C.

[131] Li Z., Zhang J., Latroche M., Wang S.M., Jiang L.J., Du J., Cuevas F. (2016). Mechanochemical synthesis under deuterium gas in the Li-Mg-N-D system: A neutron diffraction study. Phys. Chem. Chem. Phys.

[132] Li Z., Zhang J., Wang S., Jiang L., Latroche M., Du J., Cuevas F. (2015). Mechanochemistry of lithium nitride under hydrogen gas. Phys. Chem. Chem. Phys..

[133] Chen P., Xiong Z., Luo J., Lin J., Tan K.L. (2002). Interaction of hydrogen with metal nitrides and imides. Nature.

[134] Luo W. (2004). (LiNH_2_–MgH_2_): A viable hydrogen storage system. J. Alloys Compd..

[135] Matsuo M., Orimo S.-I. (2011). Lithium Fast-Ionic Conduction in Complex Hydrides: Review and Prospects. Adv. Energy Mater..

[136] Li B., Liu Y., Li C., Gao M., Pan H. (2014). In situ formation of lithium fast-ion conductors and improved hydrogen desorption properties of the LiNH_2_–MgH_2_ system with the addition of lithium halides. J. Mater. Chem. A.

[137] Li W., Wu G., Xiong Z., Feng Y.-P., Chen P. (2012). Li+ ionic conductivities and diffusion mechanisms in Li-based imides and lithium amide. Phys. Chem. Chem. Phys.

[138] Beister H.J., Haag S., Kniep R., Strössner K., Syassen K. (1988). Phase Transformations of Lithium Nitride under Pressure. Angew. Chem. Int. Ed..

[139] Bortz M., Bertheville B., Böttger G., Yvon K. (1999). Structure of the high pressure phase g-MgH_2_ by neutron powder diffraction. J. Alloys Compd..

[140] Huot J., Swainson I., Schulz R. (2006). Phase transformation in magnesium hydride induced by ball milling. Eur. J. Control.

[141] Cuevas F., Korablov D., Latroche M. (2012). Synthesis, structural and hydrogenation properties of Mg-rich MgH_2_–TiH_2_ nanocomposites prepared by reactive ball milling under hydrogen gas. Phys. Chem. Chem. Phys..

[142] Khawam A., Flanagan D.R. (2006). Solid-State Kinetic Models: Basics and Mathematical Fundamentals. J. Phys. Chem. B.

[143] Weidner E., Bull D., Shabalin I., Keens S., Telling M., Ross D., Ross K. (2007). Observation of novel phases during deuteration of lithium nitride from in situ neutron diffraction. Chem. Phys. Lett..

[144] Behrendt G., Reichert C., Kohlmann H. (2016). Hydrogenation Reaction Pathways in the Systems Li_3_N–H_2_, Li_3_N–Mg–H_2_, and Li_3_N–MgH_2_–H_2_ by in Situ X-ray Diffraction, in Situ Neutron Diffraction, and in Situ Thermal Analysis. J. Phys. Chem. C.

[145] Bull D.J., Sorbie N., Baldissin G., Moser D., Telling M.T.F., Smith R.I., Gregory D.H., Ross D.K. (2011). In situ powder neutron diffraction study of non-stoichiometric phase formation during the hydrogenation of Li_3_N. Faraday Discuss..

[146] Li Z., Qiu H.C., Wang S.M., Jiang L.J., Du J., Zhang J., Latroche M., Cuevas F. Mechanochemistry and hydrogen storage properties of 2Li_3_N + Mg mixture. Rare Met..

[147] Valiev R.Z., Estrin Y., Horita Z., Langdon T.G., Zechetbauer M.J., Zhu Y.T. (2006). Producing bulk ultrafine-grained materials by severe plastic deformation. JOM.

[148] Zhilyaev A.P., Langdon T.G. (2008). Using high-pressure torsion for metal processing: Fundamentals and applications. Prog. Mater. Sci..

[149] Kusadome Y., Ikeda K., Nakamori Y., Orimo S.-I., Horita Z. (2007). Hydrogen storage capability of MgNi_2_ processed by high pressure torsion. Scr. Mater..

[150] Leiva D.R., Jorge A.M., Ishikawa T.T., Huot J., Fruchart D., Miraglia S., Kiminami C.S., Botta W.J. (2010). Nanoscale Grain Refinement and H-Sorption Properties of MgH_2_ Processed by High-Pressure Torsion and Other Mechanical Routes. Adv. Eng. Mater..

[151] Revesz A., Kánya Z., Verebélyi T., Szabó P., Zhilyaev A., Spassov T., Zhilyaev A. (2010). The effect of high-pressure torsion on the microstructure and hydrogen absorption kinetics of ball-milled Mg_70_Ni_30_. J. Alloys Compd..

[152] Edalati K., Yamamoto A., Horita Z., Ishihara T. (2011). High-pressure torsion of pure magnesium: Evolution of mechanical properties, microstructures and hydrogen storage capacity with equivalent strain. Scr. Mater..

[153] 1De Lima G.F., Leiva D.R., Ishikawa T.T., Bolfarini C., Kiminami C.S., Botta W.J., Jorge A.M. (2011). Hydrogen sorption properties of the complex hydride Mg2FeH6 consolidated by HPT. Mater. Sci. Forum.

[154] Révész Á., Kis-Tóth Á., Varga L., Schafler E., Bakonyi I., Spassov T. (2012). Hydrogen storage of melt-spun amorphous Mg_65_Ni_20_Cu_5_Y_10_ alloy deformed by high-pressure torsion. Int. J. Hydrog. Energy.

[155] Botta W.J., Jorge Jr A.M., Veron M., Rauch E.F., Ferrie E., Yavari A.R., Huot J., Leiva D.R. (2013). H-sorption properties and structural evolution of Mg processed by severe plastic deformation. J. Alloys Compd..

[156] Zou J.X., Pérez-Brokate C.F., Arruffat R., Bolle B., Fundenberger J.J., Zeng X.Q., Grosdidier T., Ding W.J. (2014). Nanostructured bulk Mg + MgO composite synthesized through arc plasma evaporation and high pressure torsion for H-storage application. Mater. Sci. Eng. B.

[157] Hongo T., Edalati K., Arita M., Matsuda J., Akiba E., Horita Z. (2015). Significance of grain boundaries and stacking faults on hydrogen storage properties of Mg_2_Ni intermetallics processed by high-pressure torsion. Acta Mater..

[158] Grosdidier T., Fundenberger J., Zou J., Pan Y., Zeng X. (2015). Nanostructured Mg based hydrogen storage bulk materials prepared by high pressure torsion consolidation of arc plasma evaporated ultrafine powders. Int. J. Hydrog. Energy.

[159] Xu C., Lin H., Edalati K., Li W., Li L., Zhu Y. (2018). Superior hydrogenation properties in a Mg65Ce20Ni10Cu5 nanoglass processed by melt-spinning followed by high-pressure torsion. Scr. Mater..

[160] Panda S., Fundenberger J.-J., Zhao Y., Zou J., Toth L.S., Grosdidier T. (2017). Effect of initial powder type on the hydrogen storage properties of high-pressure torsion consolidated Mg. Int. J. Hydrog. Energy.

[161] Grill A., Horky J., Panigrahi A., Krexner G., Zehetbauer M. (2015). Long-term hydrogen storage in Mg and ZK60 after Severe Plastic Deformation. Int. J. Hydrog. Energy.

[162] Edalati K., Matsuda J., Iwaoka H., Toh S., Akiba E., Horita Z. (2013). High-pressure torsion of TiFe intermetallics for activation of hydrogen storage at room temperature with heterogeneous nanostructure. Int. J. Hydrog. Energy.

[163] Edalati K., Matsuda J., Arita M., Daio T., Akiba E., Horita Z. (2013). Mechanism of activation of TiFe intermetallics for hydrogen storage by severe plastic deformation using high-pressure torsion. Appl. Phys. Lett..

[164] Edalati K., Matsuda J., Yanagida A., Akiba E., Horita Z. (2014). Activation of TiFe for hydrogen storage by plastic deformation using groove rolling and high-pressure torsion: Similarities and differences. Int. J. Hydrog. Energy.

[165] Edalati K., Matsuo M., Emami H., Itano S., Alhamidi A., Staykov A., Smith D.J., Orimo S.-I., Akiba E., Horita Z. (2016). Impact of severe plastic deformation on microstructure and hydrogen storage of titanium-iron-manganese intermetallics. Scr. Mater..

[166] Edalati K., Shao H., Emami H., Iwaoka H., Akiba E., Horita Z. (2016). Activation of titanium-vanadium alloy for hydrogen storage by introduction of nanograins and edge dislocations using high-pressure torsion. Int. J. Hydrog. Energy.

[167] Edalati K., Novelli M., Itano S., Li H.-W., Akiba E., Horita Z., Grosdidier T. (2018). Effect of gradient-structure versus uniform nanostructure on hydrogen storage of Ti-V-Cr alloys: Investigation using ultrasonic SMAT and HPT processes. J. Alloys Compd..

[168] Edalati K., Uehiro R., Ikeda Y., Li H.-W., Emami H., Filinchuk Y., Arita M., Sauvage X., Tanaka I., Akiba E. (2018). Design and synthesis of a magnesium alloy for room temperature hydrogen storage. Acta Mater..

[169] Fujiwara K., Uehiro R., Edalati K., Li H.-W., Floriano R., Akiba E., Horita Z. (2018). New Mg–V–Cr BCC Alloys Synthesized by High-Pressure Torsion and Ball Milling. Mater. Trans..

[170] Edalati K., Uehiro R., Fujiwara K., Ikeda Y., Li H.-W., Sauvage X., Valiev R.Z., Akiba E., Tanaka I., Horita Z. (2017). Ultra-severe plastic deformation: Evolution of microstructure, phase transformation and hardness in immiscible magnesium-based systems. Mater. Sci. Eng. A.

[171] Edalati K., Akiba E., Horita Z. (2018). High-pressure torsion for new hydrogen storage materials. Sci. Technol. Adv. Mater..

[172] Edalati K., Daio T., Lee S., Horita Z., Nishizaki T., Akune T., Nojima T., Sasaki T. (2014). High strength and superconductivity in nanostructured niobium-titanium alloy by high-pressure torsion and annealing: Significance of elemental decomposition and supersaturation. Acta Mater..

[173] Edalati K., Emami H., Staykov A., Smith D.J., Akiba E., Horita Z. (2015). Formation of metastable phases in magnesium–titanium system by high-pressure torsion and their hydrogen storage performance. Acta Mater..

[174] Emami H., Edalati K., Staykov A., Hongo T., Iwaoka H., Horita Z., Akiba E. (2016). Solid-state reactions and hydrogen storage in magnesium mixed with various elements by high-pressure torsion: Experiments and first-principles calculations. RSC Adv..

[175] Edalati K., Emami H., Ikeda Y., Iwaoka H., Tanaka I., Akiba E., Horita Z. (2016). New nanostructured phases with reversible hydrogen storage capability in immiscible magnesium–zirconium system produced by high-pressure torsion. Acta Mater..

[176] Reilly J.J., Wiswall R.H. (1974). Formation and properties of iron titanium hydride. Inorg. Chem..

[177] Emami H., Edalati K., Matsuda J., Akiba E., Horita Z. (2015). Hydrogen storage performance of TiFe after processing by ball milling. Acta Mater..

[178] Nomura K., Uruno H., Ono S., Shinozuka H., Suda S. (1985). Effects of lattice strain on the hysteresis of pressure-composition isotherms for the LaNi_5_ H_2_ system. J. Less Common Met..

[179] Inui H., Yamamoto T., Hirota M., Yamaguchi M. (2002). Lattice defects introduced during hydrogen absorption–desorption cycles and their effects on P–C characteristics in some intermetallic compounds. J. Alloys Compd..

[180] Nagel H., Perkins R.S. (1975). Crystallographic Investigation of Ternary Titanium Vanadium Hydrides. Z. Met..

[181] Ono S., Nomura K., Ikeda Y. (1980). The raction of hydrogen with alloys of vanadium and titanium. J. Less Common Met..

[182] Hara S., Sakaki K., Itoh N., Kimura H.-M., Asami K., Inoue A. (2000). An amorphous alloy membrane without noble metals for gaseous hydrogen separation. J. Membr. Sci..

[183] Nagai H., Kitagaki K., Shoji K. (1987). Microstructure and hydriding characteristics of FeTi alloys containing manganese. J. Less Common Met..

[184] Lee S.M., Perng T.P. (1994). Effect of the 2nd phase on the initiation of hydrogenation of TiFe1-xMx (M = Cr, Mn) alloys. Int. J. Hydrog. Energy.

[185] Rigney D., Naylor M., Divakar R., Ives L. (1986). Low energy dislocation structures caused by sliding and by particle impact. Mater. Sci. Eng..

[186] Samih Y., Marcos G., Stein N., Allain N., Fleury E., Dong C., Grosdidier T. (2014). Microstructure modifications and associated hardness and corrosion improvements in the AISI 420 martensitic stainless steel treated by high current pulsed electron beam (HCPEB). Surf. Coat. Technol..

[187] Lu K., Lu J. (2004). Nanostructured surface layer on metallic materials induced by surface mechanical attrition treatment. Mater. Sci. Eng. A.

[188] Liu G., Lü J., Lu K. (2000). Surface nanocrystallization of 316L stainless steel induced by ultrasonic shot peening. Mater. Sci. Eng. A.

[189] Bagherifard S., Guagliano M. (2012). Fatigue behavior of a low-alloy steel with nanostructured surface obtained by severe shot peening. Eng. Fract. Mech..

[190] Novelli M., Fundenberger J.-J., Bocher P., Grosdidier T. (2016). On the effectiveness of surface severe plastic deformation by shot peening at cryogenic temperature. Appl. Surf. Sci..

[191] Novelli M., Bocher P., Grosdidier T. (2018). Effect of cryogenic temperatures and processing parameters on gradient-structure of a stainless steel treated by ultrasonic surface mechanical attrition treatment. Mater. Charact..

[192] Badreddine J., Rouhaud E., Micoulaut M., Remy S. (2014). Simulation of shot dynamics for ultrasonic shot peening: Effects of process parameters. Int. J. Mech. Sci..

[193] Akiba E., Iba H. (1998). Hydrogen absorption by Laves phase related BCC solid solution. Intermetallics.

[194] Yu X., Wu Z., Xia B., Xu N. (2004). The activation mechanism of Ti–V-based hydrogen storage alloys. J. Alloys Compd..

[195] Miraglia S., De Rango P., Rivoirard S., Fruchart D., Charbonnier J., Skryabina N. (2012). Hydrogen sorption properties of compounds based on BCC Ti_1_- xV_1_-yCr_1_ + x +y alloys. J. Alloys Compd..

[196] Huot J., Enoki H., Akiba E. (2008). Synthesis, phase transformation, and hydrogen storage properties of ball-milled TiV_0.9_Mn_1.1_. J. Alloys Compd..

[197] Matsuda J., Akiba E. (2013). Lattice defects in V–Ti BCC alloys before and after hydrogenation. J. Alloys Compd..

[198] Kim H., Sakaki K., Ogawa H., Nakamura Y., Nakamura J., Akiba E., Machida A., Watanuki T., Proffen T. (2013). Origin of Degradation in the Reversible Hydrogen Storage Capacity of V1–xTixAlloys from the Atomic Pair Distribution Function Analysis. J. Phys. Chem. C.

[199] Huot J. (2016). Enhancing Hydrogen Storage Properties of Metal Hydrides.

[200] Ueda T.T., Tsukahara M., Kamiya Y., Kikuchi S. (2005). Preparation and hydrogen storage properties of Mg–Ni–Mg_2_Ni laminate composites. J. Alloys Compd..

[201] Dufour J., Huot J. (2007). Rapid activation, enhanced hydrogen sorption kinetics and air resistance in laminated Mg–Pd 2.5at.%. J. Alloys Compd..

[202] Dufour J., Huot J. (2007). Study of Mg_6_Pd alloy synthesized by cold rolling. J. Alloys Compd..

[203] Løken S., Solberg J., Maehlen J., Denys R., Lototsky M., Tarasov B., Yartys V., Denys R. (2007). Nanostructured Mg–Mm–Ni hydrogen storage alloy: Structure–properties relationship. J. Alloys Compd..

[204] Mori R., Miyamura H., Kikuchi S., Tanaka K., Takeichi N., Tanaka H., Kuriyama N., Ueda T.T., Tsukahara M. (2007). Hydrogenation Characteristics of Mg Based Alloy Prepared by Super Lamination Technique. Mater. Sci. Forum.

[205] Suganuma K., Miyamura H., Kikuchi S., Takeichi N., Tanaka K., Tanaka H., Kuriyama N., Ueda T.T., Tsukahara M. (2007). Hydrogen Storage Properties of Mg-Al Alloy Prepared by Super Lamination Technique. Adv. Mater. Res..

[206] Takeichi N., Tanaka K., Tanaka H., Ueda T.T., Kamiya Y., Tsukahara M., Miyamura H., Kikuchi S. (2007). The hydrogen storage properties of Mg/Cu and Mg/Pd laminate composites and metallographic structure. J. Alloys Compd..

[207] Takeichi N., Tanaka K., Tanaka H., Ueda T.T., Tsukahara M., Miyamura H., Kikuchi S. (2007). Hydrogen Storage Properties and Corresponding Phase Transformations of Mg/Pd Laminate Composites Prepared by a Repetitive-Rolling Method. Mater. Trans..

[208] Ebrahimi-Purkani A., Kashani-Bozorg S. (2008). Nanocrystalline Mg2Ni-based powders produced by high-energy ball milling and subsequent annealing. J. Alloys Compd..

[209] Pedneault S., Huot J., Roué L. (2008). Nanostructured Mg2Ni materials prepared by cold rolling and used as negative electrode for Ni–MH batteries. J. Power Sources.

[210] Pedneault S., Roué L., Huot J. (2008). Synthesis of Metal Hydrides by Cold Rolling. Mater. Sci. Forum.

[211] Danaie M., Mauer C., Mitlin D., Huot J. (2011). Hydrogen storage in bulk Mg–Ti and Mg–stainless steel multilayer composites synthesized via accumulative roll-bonding (ARB). Int. J. Hydrog. Energy.

[212] Lang J., Huot J. (2011). A new approach to the processing of metal hydrides. J. Alloys Compd..

[213] Leiva D., Floriano R., Huot J., Jorge A., Bolfarini C., Kiminami C., Ishikawa T., Botta W., Leiva D., Kiminami C. (2011). Nanostructured MgH_2_ prepared by cold rolling and cold forging. J. Alloys Compd..

[214] Amira S., Huot J. (2012). Effect of cold rolling on hydrogen sorption properties of die-cast and as-cast magnesium alloys. J. Alloys Compd..

[215] Bellemare J., Huot J. (2012). Hydrogen storage properties of cold rolled magnesium hydrides with oxides catalysts. J. Alloys Compd..

[216] Floriano R., Leiva D.R., Deledda S., Hauback B.C., Botta W.J. (2013). Nanostructured MgH_2_ Obtained by Cold Rolling Combined with Short-time High-energy Ball Milling. Mater. Res. Ibero am. J. Mater..

[217] Floriano R., Leiva D., Deledda S., Hauback B., Botta W., Leiva D., Botta W. (2013). Cold rolling of MgH_2_ powders containing different additives. Int. J. Hydrog. Energy.

[218] Zhang H., Huang G., Wang L., Roven H.J., Pan F. (2013). Enhanced mechanical properties of AZ31 magnesium alloy sheets processed by three-directional rolling. J. Alloys Compd..

[219] Floriano R., Leiva D., Deledda S., Hauback B., Botta W., Botta W. (2014). MgH_2_-based nanocomposites prepared by short-time high energy ball milling followed by cold rolling: A new processing route. Int. J. Hydrog. Energy.

[220] Asselli A., Leiva D., Huot J., Kawasaki M., Langdon T., Botta W. (2015). Effects of equal-channel angular pressing and accumulative roll-bonding on hydrogen storage properties of a commercial ZK60 magnesium alloy. Int. J. Hydrog. Energy.

[221] Faisal M., Gupta A., Shervani S., Balani K., Subramaniam A. (2015). Enhanced hydrogen storage in accumulative roll bonded Mg-based hybrid. Int. J. Hydrog. Energy.

[222] Li Q., Lin Q., Chou K.-C., Jiang L.-J., Zhan F. (2004). Hydrogen storage properties of mechanically alloyed Mg–8 mol% LaNi0.5 composite. J. Mater. Res..

[223] Liu D., Liu X., Zhu Y., Li L. (2008). Hydriding combustion synthesis of Mg–CaNi_5_ composites. J. Alloys Compd..

[224] Soyama J., Floriano R., Leiva D.R., Guo Y., Jorge Junior A.M., Pereira da Silva E., Pinto H.C., Bolfarini C., Kiminami C.S., Botta W.J. (2016). Severely deformed ZK60 + 2.5% Mm alloy for hydrogen storage produced by two different processing routes. Int. J. Hydrog. Energy.

[225] Floriano R., Leiva D., Melo G., Ishikawa T., Huot J., Kaufman M., Figueroa S., Mendoza-Zélis L., Damonte L., Botta W. (2017). Low temperature rolling of AZ91 alloy for hydrogen storage. Int. J. Hydrog. Energy.

[226] Jung J.Y., Fadonougbo J.O., Suh J.-Y., Lee Y.-S., Huh J.-Y., Cho Y.W. (2018). Synthesis of Mg2FeH6 by hydrogenation of Mg/Fe powder mixture prepared by cold roll milling in air: Effects of microstructure and oxygen distribution. Int. J. Hydrog. Energy.

[227] Márquez J.J., Leiva D.R., Floriano R., Soyama J., Silva W.B., Ishikawa T.T., Kiminami C.S., Botta W.J. (2018). Hydrogen storage in MgH 2 LaNi 5 composites prepared by cold rolling under inert atmosphere. Int. J. Hydrog. Energy.

[228] Yoo M.H. (1981). Slip, twinning, and fracture in hexagonal close-packed metals. Met. Mater. Trans. A.

[229] Tonda H., Ando S. (2002). Effect of temperature and shear direction on yield stress by {1122}<1123> slip in HCP metals. Met. Mat Trans A.

[230] Broom D.P. (2011). Hydrogen Storage Materials: The Characterisation of Their Storage Properties.

[231] Vucht J.H.N.V., Kuijpers F.A., Bruning H.C.A.M. (1970). Reversible room-temperature absorption of large quantities of hydrogen by intermetallic compounds. Philips Res. Rep..

[232] Hanada N., Nakagawa T., Asada H., Ishida M., Takahashi K., Isobe S., Saita I., Asano K., Nakamura Y., Fujisawa A. (2015). Dependence of constituent elements of AB 5 type metal hydrides on hydrogenation degradation by CO_2_ poisoning. J. Alloys Compd..

[233] Seta S., Uchida H. (1995). Hydrogen solubility in LaNi_5_. J. Alloys Compd..

[234] Termsuksawad P., Niyomsoan S., Mishra B., Olson D., Gavra Z., Kaydanov V. (2005). Prediction of hydrogen absorption behavior in AB5 hydrogen storage alloys by electronic techniques. Mater. Sci. Eng. B.

[235] Uchida H., Sato M., Moriwaki O. (1997). Hydrogen absorption and desorption isotherms in the solid solution regions of the LaNi_5_–H system. J. Alloys Compd..

[236] Tousignant M., Huot J. (2014). Hydrogen sorption enhancement in cold rolled LaNi_5_. J. Alloys Compd..

[237] Huot J., Tousignant M. (2017). Hydrogen sorption enhancement in cold-rolled and ball-milled CaNi_5_. J. Mater. Sci..

[238] Yoshikawa A., Uyenishi Y., Iizumi H., Matsumoto T., Takano N., Terasaki F. (1998). Determination of the positionand occupancy of deuterium in CaNi_5_ deuterides by neutron diffraction. J. Alloys Compd..

[239] Couillaud S., Enoki H., Amira S., Bobet J., Akiba E., Huot J. (2009). Effect of ball milling and cold rolling on hydrogen storage properties of nanocrystalline TiV1.6Mn0.4 alloy. J. Alloys Compd..

[240] Dupim I., Moreira J., Huot J., Santos S. (2015). Effect of cold rolling on the hydrogen absorption and desorption kinetics of Zircaloy-4. Mater. Chem. Phys..

[241] Khajavi S., Rajabi M., Huot J. (2019). Effect of cold rolling and ball milling on first hydrogenation of Ti_0.5_Zr_0_._5_ (Mn_1-x_Fe_x_) Cr_1_, x = 0, 0.2, 0.4. J. Alloys Compd..

[242] Leiva D.R., Costa H.C.D.A., Huot J., Pinheiro T.S., Junior A.M.J., Ishikawa T.T., Filho W.J.B. (2012). Magnesium-Nickel alloy for hydrogen storage produced by melt spinning followed by cold rolling. Mater. Res..

[243] Lima G.F., Triques M.R.M., Kiminami C.S., Botta W.J., Jorge Jr A.M. (2014). Hydrogen storage properties of 2Mg–Fe after the combined processes of hot extrusion and cold rolling. J. Alloys Compd..

[244] Friščić T., Halasz I., Beldon P.J., Belenguer A.M., Adams F., Kimber S.A.J., Honkimäki V., Dinnebier R.E. (2013). Real-time and in situ monitoring of mechanochemical milling reactions. Nat. Chem..

[245] Halasz I., Kimber S.A.J., Beldon P.J., Belenguer A.M., Adams F., Honkimäki V., Nightingale R.C., E Dinnebier R., Friščić T. (2013). In situ and real-time monitoring of mechanochemical milling reactions using synchrotron X-ray diffraction. Nat. Protoc..

[246] Katsenis A.D., Puškarić A., Strukil V., Mottillo C., Julien P.A., Uzarevic K., Pham M.-H., Do T.-O., Kimber S.A.J., Lazić P. (2015). In situ X-ray diffraction monitoring of a mechanochemical reaction reveals a unique topology metal-organic framework. Nat. Commun..

[247] Filinchuk Y., Ban V., Morelle F., Tumanov N., Dovgaliuk I., Cerny R., Sadikin Y., Schouwink P. Mechanochemical synthesis of hydrides followed in situ by X-ray diffraction. Proceedings of the 14th International Symposium on Metal-Hydrogen Systems: Fundamentals and Applications (MH2014).

[248] Batzdorf L., Fischer F., Wilke M., Wenzel K.-J., Emmerling F. (2015). Direct in situ investigation of milling reactions using combined x-ray diffraction and raman spectroscopy. Angew. Chem. Int. Ed..

[249] Halasz I., Užarević K., Friščić T. (2015). Real-Time and in Situ Monitoring of Mechanochemical Reactions: A New Playground for All Chemists. J. Phys. Chem. Lett..

[250] Halasz I., Puškarić A., Kimber S.A.J., Beldon P.J., Belenguer A.M., Adams F., Honkimäki V., Dinnebier R.E., Patel B., Jones W. (2013). Real-Time in Situ Powder X-ray Diffraction Monitoring of Mechanochemical Synthesis of Pharmaceutical Cocrystals. Angew. Chem..

[251] Fischer F., Heidrich A., Greiser S., Benemann S., Rademann K., Emmerling F. (2016). Polymorphism of Mechanochemically Synthesized Cocrystals: A Case Study. Cryst. Growth Des..

[252] Fischer F., Lubjuhn D., Greiser S., Rademann K., Emmerling F. (2016). Supply and Demand in the Ball Mill: Competitive Cocrystal Reactions. Cryst. Growth Des..

[253] Gracin D., Štrukil V., Friščić T., Halasz I., Užarević K. (2014). Laboratory Real-Time and in Situ Monitoring of Mechanochemical Milling Reactions by Raman Spectroscopy. Angew. Chem..

[254] Kulla H., Haferkamp S., Akhmetova I., Röllig M., Maierhofer C., Rademann K., Emmerling F.L. (2018). In Situ Investigations of Mechanochemical One-Pot Syntheses. Angew. Chem. Int. Ed..

[255] Tumanov N., Ban V., Poulain A., Filinchuk Y. (2017). 3D-printed jars for ball-milling experiments monitored in situ by X-ray powder diffraction. J. Appl. Crystallogr..

[256] Ban V., Sadikin Y., Lange M., Tumanov N., Filinchuk Y., Černý R., Casati N. (2017). Innovative in Situ Ball Mill for X-ray Diffraction. Anal. Chem..

